# A semi purified hydroalcoholic fraction from *Caesalpinia bonduc* seeds causes ergosterol biosynthesis inhibition in *Candida albicans* resulting in cell membrane damage

**DOI:** 10.3389/fphar.2023.1189241

**Published:** 2023-06-12

**Authors:** Shan Sasidharan, Kumar S. Nishanth, Hareendran. J Nair

**Affiliations:** 1Department of R&D, Pankajakasthuri Herbal Research Foundation, Pankajakasthuri Ayurveda Medical College Campus, Trivandrum, Kerala, India; 2Department of R&D, Pankajakasthuri Herbals India Pvt Ltd., Pankajakasthuri Ayurveda Medical College Campus, Trivandrum, Kerala, India

**Keywords:** *C. bonduc*, *Candida albicans*, ergosterol, molecular docking, antifungal

## Abstract

*Candida* species are currently developing resistance to prevailing commercially available drugs, which raises an instantaneous need to discover novel antifungals. To cope with this shocking situation, phytochemicals are the richest, safest, and most potent source of excellent antimicrobials with broad-spectrum activity. The aim of the current study is to explore the anticandidal potential of the various fractions purified from the hydroalcoholic extract of *C. bonduc* seed. Out of five fractions purified from the hydroalcoholic extract, fraction 3 (Fr. 3) recorded the best activity against *C. albicans* (8 μg/mL) and thus this species was chosen for further mechanism of action studies. The phytochemical examination reveals that Fr. 3 was found to contain steroids and triterpenoids. This was further supported by LC-QTOF-MS and GCMS analyses. Our findings show that Fr. 3 targets the ergosterol biosynthesis pathway in *C. albicans* by inhibiting the lanosterol 14-α demethylase enzyme and downregulating expression of its related gene ERG11. Molecular docking outcomes disclosed favorable structural dynamics of the compounds, implying that the compounds present in Fr. 3 would be able to successfully bind to the lanosterol 14-α demethylase, as evidenced by the docked compounds’ strong interaction with the target enzyme’s amino acid residues. Considering virulence factors, the Fr. 3 recorded significant antibiofilm activity as well as germ-tube reduction potential. Furthermore, Fr. 3 enhances the production of intracellular reactive oxygen species (ROS). This suggests that the antifungal activity of Fr. 3 was associated with membrane damage and the induction of ROS production, resulting in cell death. Fluorescence microscopic analysis of PI stained *Candida* further showed changes in the plasma membrane permeability, which causes severe loss of intracellular material and osmotic balance. This was demonstrated by the potassium ion leakage and release of genetic materials. Finally, the erythrocyte lysis assay confirmed the low cytotoxicity of Fr. 3. Both *in silico* and *in vitro* results suggest that Fr. 3 has the potential to propel forward novel antifungal drug discovery programmes.

## Introduction

Infections associated with pathogenic fungi are a persistent and solemn threat to humans, particularly in immunocompromised individuals, where the occurrence of systemic candidiasis caused by various *Candida* species has augmented significantly in recent years (Morais et al., 2021; Contreras Martínez et al., 2022). The genus *Candida* contains more than 200 species, out of which only a few are human pathogenic organisms, and they all fall under the category, opportunistic pathogens. *Candida albicans*, *Candida glabrata*, *Candida parapsilosis*, *Candida tropicalis*, and *Candida krusei* account for about 90% of all identifiable invasive *Candida* infections affecting humans. Out of the five above mentioned *Candida* species, C. *albicans* is the most prevalent cause of human fungal infections which is one of major cause of severe fungal intra-hospital infections in various susceptible patients (Stojanović-Radić et al., 2020).

Antifungal drugs such as echinocandins, azoles, and polyenes are currently used to treat candidiasis. These drugs either inhibit beta-1,3-D glucan synthesis in the fungal cell wall or disrupt the ergosterol biosynthesis pathway in the cell membrane. Regrettably, existing antifungal drug classes have limitations such as high toxicity, limited spectrum activity, and exorbitant prices (Patel et al., 2020). Despite extensive research to discover novel antifungal drugs, the usually available drugs for the treatment of candidiasis are somewhat limited since fungi are also eukaryotes. In addition to this, the appearance of drug-resistant *Candida* strains is very common due to the widespread and prolonged use of antifungal drugs (Lee and Kim, 2022). Widespread and recurrent use of current antifungal agents, particularly azole drugs, has led to the rapid incidence of antifungal resistance, especially among the pathogenic *Candida* species (Tong et al., 2021).

*Candida* species are eukaryotic organisms with a high degree of genetic similarity with humans. Hence, it is extremely difficult to target a unique drug target specific to fungal cells that human cells lack. Due to this situation, ergosterol and its biosynthesis pathway are one of these unique targets. Hence, targeting the ergosterol biosynthesis pathway has become a prevalent strategy for developing novel antifungals, including azoles and polyene derivatives (Mazu et al., 2016). But, the majority of these antifungal medications from various classes are connected with a side effect, including several toxicities. Prolonged use of amphotericin B affects the functions of the kidney and liver and further leads to infusion-related reactions as well as hypokalemia, whereas flucytosine has been linked to anaemia and neutropenia. Except for fluconazole, all azole drugs are reported to cause hematopoiesis and to further inhibit macrophage colony-forming units in the bone marrow of humans. Furthermore, these drug classes have been linked to narrow spectrum and drug resistance (Rather et al., 2022). Due to this, it is critical to find new antifungal agents that are both safe and effective in managing various *Candida* infections. Natural products and their derivatives are well reported to play a vital role in pharmacological research that leads to the discovery of novel therapeutic agents in this scenario. In this context, medicinal plants are a rich source of novel pharmacologically active compounds for managing severe fungal diseases (Medeiros et al., 2020). Novel phytochemicals provide an excellent source of chemical scaffolds with a wide range of biological activities that are useful in drug development (Harvey et al., 2015).

*Caesalpinia bonduc* (L.) Roxb. is also known as *C. bonducella* (L.) Fleming [Fabaceae (Caesalpinaceae)]. This plant is also known as “fever nut,” “bonduc nut,” and “nicker nut.” Various herbal preparations are made from all parts of this plant, including the roots, leaves, seeds, bark, and stem. It has been reported that the seeds of *C. bonduc* have antidiarrhoeal, antiviral, antibacterial, antimicrobial, antifungal, antidiabetic, antitumor, antipyretic and analgesic properties, as well as trypsin and chymotrypsin inhibitor properties (Billah et al., 2013). Recently, we have investigated the anticandidal activity of the various seed extracts of *C. bonduc* against four *Candida* species. In this study, alcoholic followed by hydroalcoholic extract recorded significant activity against *Candida* species (Sasidharan et al., 2022a).

The primary aim of this investigation was to test the antifungal activity of various fractions purified from a hydroalcoholic extract of *C. bonduc* seeds *in vitro*. Here, Fr. 3 demonstrated significant antifungal activity, hence subjected to LC-Q-TOF-MS analysis to determine the active metabolites present in this fraction. Following an initial screening, we assessed Fr. 3’s antibiofilm property against *C. albicans*, followed by its effects on ergosterol inhibition. Following that, we performed molecular docking studies against the lanosterol 14-α-demethylase enzyme, a key target in the biosynthesis of ergosterol. We assessed the response to Fr. 3 treatment by looking at the expression of a gene (ERG 11) involved in ergosterol biosynthesis. A more in-depth investigation of the mechanisms of action of Fr. 3 was conducted, beginning with the aggregation of intracellular reactive oxygen species (ROS), followed by membrane integrity and permeability. Finally, we conducted experiments to confirm cellular content leakage through Fr. 3 treated C. albicans damaged membrane.

## Materials and methods

### Collection and authentication of *C. bonduc* seeds

The *C. bonduc* seeds were collected in and around the Ernakulum region (9.98°N 76.28°E), Kerala, India, between January and April 2019. The pharmacognostic identification of the Caesalpinia bonduc seeds, including macroscopic, microscopic and organoleptic assessment were conducted as per the details provided in the Ayurvedic Pharmacopoeia of India (API, 2016). Authentication and validation of the seeds were done by Dr. Dan Mathew, Principal Scientist at JNTBGRI, Trivandrum, Kerala, India. The specimen of the seeds was deposited in the Herbarium of the Department of Medicinal Plants, Pankajakasthuri Ayurveda Medical College and PG Centre, Killy, Kattakada, Trivandrum, Kerala, India, vide voucher number (PKMAC VC/IS-035).

### Plant material preparation

The collected seeds were washed with tap water to remove debris and then dried in a hot air oven at 40°C until constant weight. Once fully dried, the seeds were grounded by mechanical milling until a fine powder was obtained (Sasidharan et al., 2022b).

### Preparation of hydroalcoholic extract of *C. bonduc* seeds

Powdered seed material (2 kg) was subjected to cold maceration with 50% alcohol: water in (4 L) and was stirred every 8 h periodically, for 3 days. After the completion of the extraction period, initial filtration of the macerate was done through a filtration cloth to obtain the supernatant, separated from the macerated powder. This supernatant was subjected to subsequent filtration by passing it through a Buchner funnel assembly and Whatman filter paper number 1 under reduced pressure. This secondary filtration removed solid particulate matter suspended in the filtrate. Finally, a rotary evaporator was used for the evaporation of methanol from pure filtrate at 40°C under reduced pressure which yielded a dark brown gummy mass. The left-over solvents in the extracts were thoroughly removed using a vacuum oven. Finally, the extracts were subjected to freeze-drying using a lyophilizer and stored at 4°C for future studies.

### Fractionation of hydroalcoholic extract

The hydroalcoholic extract of *C. bonduc* seeds was subjected by silica gel column chromatography (45 cm × 5 cm in size). The hydroalcoholic seed extract (30 gm) was loaded on top of the glass column packed with silica gel (100–200 mesh). After that, the column was eluted with different proportions of petroleum ether, ethyl acetate, and ethanol. Initial, the elution was performed with 100% petroleum ether, followed by a combination of petroleum ether and ethyl acetate in ratios of 4:1, 3:2, 1:1, 2:3 and 1:4 followed by ethyl acetate: methanol combination in the ratios of 10:0, 9:1, 7:3, 5:5, 3:7 and 0:10. Forty two fractions of 100 mL each were collected, which was further analyzed by Thin Layer Chromatography (TLC).

### Phytochemical screening of Fr. 3

The purified fractions were phytochemically evaluated to determine the presence of alkaloids, flavonoids, terpenoids, steroids according to standard methods (Harborne, 1973). Any change in colour or precipitate formation was used as an indicator of a positive response to these tests.

### Testing for alkaloids

Fr.3 (5 mg) was dissolved in 1 mL of 5% hydrochloric acid and after mixing and filtering, three aliquots were taken. Drops of Wagner, Mayer, Bouchardat and Dragendorff reagents were added to each. A red-brown precipitate (Wagner), yellowish-white precipitate (Mayer), brown precipitate (Bouchardat) and red–orange precipitate (Dragendorff) indicated the presence of such metabolites.

### Testing for flavonoids

Shinoda test. 1 mL of absolute ethanol and 3 drops of concentrated hydrochloric acid were added to 0.5 mL of diluted Fr. 3 in isopropyl alcohol. The formation of a red color indicated the presence of aurones and chalcones. In cases where no colour change was observed, pieces of metallic magnesium were added. The formation of orange, red or magenta coloration indicated the presence of flavones and flavonols, respectively.

### Testing for steroids and/or triterpenoids

Salkowski test. 2 mL of chloroform and 1 mL of concentrated sulfuric acid were slowly added to 10 drops of Fr. 3 dissolved in isopropyl alcohol until double phase formation. The presence of a dish-brown color in the middle layer was indicative of a steroidal ring.

Lieberman Bouchard test. 1 mL of anhydrous acetic acid and 3 drops of concentrated sulfuric acid were added to 2 mL of Fr.3 dissolved in isopropyl alcohol. After 5 min a blue-green color middle layer was indicative of sterols, but pink, red, magenta or violet color revealed the presence of terpenoids.

### Culture media

For antifungal studies, sabouraud dextrose broth (SDB), sabouraud dextrose agar (SDA) and yeast extract peptone dextrose (YPD) were purchased from Hi-Media (Mumbai, India), and RPMI-1640-L-glutamine (without sodium bicarbonate) (Sigma-Aldrich, India) culture media were used. They were prepared and used in accordance with the manufacturer’s directions.

### Test *Candida* strains and culture condition

Three Candida species were employed in the present study: C. albicans (ATCC 66027), *C. tropicalis* (ATCC 750), and *C. parapsilosis* (ATCC 22019). SDA agar plates containing additional 1% agar were used to maintain the cultures. Before experiments, a single colony of each *Candida* species was inoculated into SDB medium and then incubated at 30°C in a shaking incubator at 150 rpm. As small quality of culture was again transferred into a fresh SDB and incubated for 3 h. To perform all *in vitro* assays, a 3 h culture with 0.1 optical density (OD) (1 × 106 CFU/mL) was used as the inoculum.

### Minimum inhibitory concentration (MIC)/minimum fungicidal concentration (MFC)

The MIC and MFC of pooled column fractions and the standard drug (clotrimazole) were assessed by the microbroth dilution method using ELISA plates bestowed by the Clinical and Laboratory Standards Institute (Method M27-A3 (CLSI, 2008) against the three test *Candida* species. The inoculum was made by dilution of the colonies in 0.85% sterile physiological saline solution at 0.5 McFarland concentration. A spectrophotometric reading at 530 nm confirmed the concentration even further. Twofold serial dilutions of fractions were performed in RPMI 1640 medium to obtain the final concentrations that ranged from 2 to 8,000 μg/mL for Fr. 3 and from 0.250 to 512 μg/mL for clotrimazole. The experiment was conducted in a final volume of 200 µL per well as follows: culture medium containing test compounds (100 µL) and *Candida* inoculum at a concentration of 1 × 106 CFU/mL (100 µL). A positive (without test compounds) and a negative control (medium only) were also included in each set of experiments. The plate was incubated at 30°C for 24 h. The experiment was conducted in triplicate set. The minimum inhibitory concentration (MIC) was determined as the lowest concentration in the test sample that revealed no visible fungal growth.

For finding the MFC value, 100 µL from each ELISA well without visible fungal growth was directly inoculated on SDA plates. After incubation for 48 h at 30°C, the colony count was counted and expressed as CFU/mL. The MFC was defined as the minimal concentration of test sample required to kill 99.9% of the organisms (Turecka et al., 2018).

In MIC/MFC studies, Fr. 3 recorded significant anticandidal activity and thus this fraction was selected for further detailed studies, including the mechanism of action.

### Determination of bioactive compounds present in the active fraction

The characterization of bioactive phytochemicals present in the Fr. 3 was investigated using LC-QTOF-MS (Agilent Technologies, Santa Clara, CA, United States, Model no: 6545 QTOF) (Sasidharan et al., 2022c).

### Gas chromatography–mass spectrometry (GC-MS) analysis

Before going to GCMS, the Fr. 3 was derivatized by the following method: Briefly, about 50 µL pyridine, 100 µL of methoxyamine HCl (20 mg/mL in pyridine), and 300 µL of MSTFA were added to the extract. The extract solutions were filtered and covered with aluminum foil to be left overnight at room temperature before the analysis. GC–MS analyses were carried out using the Perkin-Elmer Clarus 680 system (Perkin-Elmer, Inc. United States) equipped with a fused silica column, packed with Elite-5MS) capillary column (30 m in length × 250 μm in diameter × 0.25 μm in thickness). Pure helium gas (99.99%) was used as the carrier gas at a constant flow rate of 1 mL/min. For GC–MS spectral detection, an electron ionization energy method was adopted with a high ionization energy of 70 eV (electron Volts) with 0.2 s of scan time and fragments ranging from 40 to 600 m/z. The injection quantity of 1 μL was used (split ratio 10:1), and the injector temperature was maintained at 250°C (constant). The column oven temperature was set at 50°C for 3 min, raised at 10°C per min up to 280°C, and final temperature was increased to 300°C for 10 min. The contents of phytochemicals present in the test samples were identified based on comparison of their retention time (min), peak area, peak height and mass spectral patterns with those spectral databases of authentic compounds stored in the National Institute of Standards and Technology (NIST) library.

### Quantitative assessment of biofilm formation

*C. albicans* was assessed to quantify the lessening of biofilms when treated with Fr. 3 by adopting the protocol reported by Donadu et al. (2021), with slight amendments. For biofilm formation, *C. albicans* colonies were inoculated in YPD medium and then incubated for 24 h at 30°C to standardize the inoculum until it reached a concentration of 106 cells/mL. The fungal inoculum was then cultured in each well of 96-well plates in YPD broth and incubated at 30°C for 48 h. After that, the YPD medium was carefully removed from the plates and 200 µL of Fr. 3 at various concentrations (0.5X MIC, MIC and 2X MIC of) in YPD medium was added and incubated for 2 h at 30°C. After that, the detached C. albicans cells were removed and the biofilm formed at the bottom of the wells was washed with sterile deionized water three times. Cultures lacking Fr. 3 served as controls, and clotrimazole served as a positive control. Biofilm reductions were measured by staining wells for 15 min with 0.1% crystal violet. The samples were washed with sterile, deionized water to remove any excess dye. Finally, the samples were immersed in 250 µL of 30% glacial acetic acid. A microplate reader was used to measure absorbance (OD 590). The experiment was carried out in triplicate. The reduction potential of Fr. 3 in biofilm was determined using the formula:

$$Biofilm\;reduction\;\left(\%\right):\frac{AbsCO-AbsFr.3}{AbsCO}\times 100$$

Where AbsCO = absorbance of the control sample and Abs Fr. 3 = absorbance samples treated with Fr. 3.

### Germ-tube production

Fr. 3 was assessed for its impact on the germ tubes germination by *C. albicans*. In a sterile tube, RPMI-1640 (1 mL) medium was inoculated with a sufficient volume of the earlier prepared *Candida* suspensions in order to achieve a final concentration of 5 × 105 CFU/mL. After that, various concentrations of Fr. 3 (0.5X MIC, MIC and 2X MIC) were added to the medium and incubated at 30°C for 2 h. The cells incubated with various concentrations of Fr. 3 were counted using a hemocytometer (Alem et al., 2006). Here, the total *Candida* cell number and the number of *Candida* cells with germ tubes were estimated for each sample. The experiment was conducted in triplicate for each treatment.

### Ergosterol biosynthesis assay

To find the impact of Fr. 3 on ergosterol biosynthesis, various concentrations (0.5X MIC, MIC, and 2X MIC) of Fr. 3 were screened in *C. albicans* based on a spectrophotometric method, as reported earlier (Li et al., 2016).

In brief, a single colony of C. albicans from an SDA plate was inoculated into 20 mL of SDB containing Fr. 3 at various concentrations. In this study, plain SDB served as a control, while clotrimazole served as a positive control. The cultures were incubated in a shaking incubator at 30°C for 16 h. *C. albicans* cells in stationary phase were collected by centrifugation at 3,000 rpm for 5 min and thoroughly washed with sterile distilled water. The net wet weight of the cell pellet was then determined. Each pellet received 3 mL of a 25% alcoholic potassium hydroxide solution (25 g of KOH and 35 mL of sterile distilled water; make up to 100 ml with 100% ethanol) and was mixed for 1 minute. Cell suspensions were then transferred to sterile borosilicate glass screw-cap tubes and incubated for 1 h in an 80°C water bath. After that, the tubes were allowed to cool to room temperature. Sterols were then extracted using a 1:3 mixture of sterile distilled and n-heptane, followed by 3 min of vigorous vortexing. Then the n-heptane layer was carefully collected in a clean borosilicate glass screw-cap tube and stored at −20°C. A 1-mL aliquot of sterol extract was diluted fivefold in 100% ethanol before being scanned spectrophotometrically (Labomed, INC Spectrophotometer, United States) between 240 and 300 nm.

$$\%\;ergosterol+\%\;24\left(28\right)\;DHE=\frac{\left[\left(\frac{A281.2}{290}\right)\times F\right]}{Cell\;pellet\;weight}$$

$$\%\;24\left(28\right)\;DHE=\frac{\left[\left(\frac{A230}{518}\right)\times F\right]}{Cell\;pellet\;weight}$$

$$\%\;ergosterol=\left[\%\;ergosterol+\%\;24\left(28\right)\;DHE\right]-\%\;24\left(28\right)\;DHE$$

where F represents the ethanol dilution factor and 290 and 518 represent the E values (in percentages per cm) determined for crystalline ergosterol and 24 (28) DHE, respectively.

### Quantitation of gene expression levels

Gene expression levels of ERG11 (GenBank accession number X13296) were determined using quantitative real-time RT-PCR according to the protocol reported earlier in *C. albicans* cells treated with Fr. 3 at different concentrations (Alizadeh et al., 2018). In brief, M-MuLV reverse transcriptase and random hexamer oligonucleotides were used to make single-stranded cDNA from 0.3 µg RNA extract (Fermentas, United States). Table 1 contains a list of oligonucleotide primers. The ERG11 and actin primers were amplified using a PCR (Bio-Rad MiniOpticonTM system, United States) using TMSYBR Green qPCR Master Mix (Fermentas, United States) according to the manufacturer’s protocol. The Pfaffl method was employed to analyze relative gene expression data. Clotrimazole was used as a standard drug control.

**TABLE 1:** The sequence of oligonucleotide primers employed in the PCR.

Primer type	Orientation	Sequence
ERG11	Forward	5′TGG AGA CGT GAT GCT G 3′
	Reverse	5′AGT ATG TTG ACC ACC CAT AA3′
ACTIN	Forward	5′GAG TTG CTC CAG AAG AAC ATC CAG 3′
	Reverse	5′TGA GTA ACA CCA TCA CCA GAA TCC 3′

### Molecular docking analysis

The X-Ray crystallographic 3D protein structure of ERG 11 was retrieved from the Protein Data Bank (RCSB PDB). For proteins not available in the Protein Data Bank, the structure was modelled using Swiss-Model using homology modelling and downloaded in PDB file format. The software Biovia Discovery Studio 2021 was used to visualize and clean the proteins to remove native ligands, heteroatoms, and water molecules. The proteins were then prepared to add hydrogens and missing residues and then energy minimization was performed. The largest receptor cavity was selected as the binding site and the LibDock module was chosen to perform docking. Itraconazole, Ketoconazole, Miconazole and Fluconazole were used as the standard antifungal agents for molecular docking (Supplementary file).

### Analysis of ROS production

Endogenous levels of ROS were measured using DCFH-DA stained *Candida* cells with the aid of a fluorescence microscope. In brief, the cell density of C. albicans was adjusted to 1 × 106 cells/mL in YPD broth and subjected to various concentrations of Fr. 3 for 4 h at 30°C. At the same conditions, the positive control was treated with hydrogen peroxide (2 mmol/L). After treatment, the *C. albicans* cells were collected by centrifugation for 2,500 rpm and washed three times with PBS. Then the cells were stained with 10 mol/L DCFH-DA for 30 min at 30°C. The fluorescence was then quantified using a spectrofluorometer (Shimadzu UV-1700, Shimadzu, Kyoto, Japan) at 495 nm excitation and 525 nm emission wavelengths. Furthermore, ROS-stained C. albicans were analysed using a fluorescence microscope (BD PathwayTM Bioimager system, United States).

### Analysis of cell membrane integrity

An amended version of the protocol described by Li et al. (2016) was used to assess cell membrane integrity. Specifically, after *C. albicans* cells were treated with various concentrations of Fr. 3, they were collected and stained for 30 min in darkness with propidium iodide (PI) (10 mg/mL). The integrity of the cell membrane was then examined using a fluorescence microscope (BD PathwayTM Bioimager system, United States). The fluorescence was then measured with a spectrofluorometer (Shimadzu UV-1700, Shimadzu, Kyoto, Japan) at 488 nm excitation and 630 nm emission wavelengths. The experiment was conducted in triplicate.

### Analysis of cell membrane potential

The potential *Candida* cell membrane after treatment with Fr. 3 was assessed using the Rho 123 (rhodamine) fluorescence method (Zhang et al., 2016). Briefly *C. albicans* was inoculated to YPD and incubated for approximately 15 h and then adjusted the cell density to 1 × 106 CFU/mL. Then Fr. 3 at 0.5X, MIC, and 2X MIC concentration was added and incubated at 30°C for 3 h. After completion of the incubation period, dissolving Rho 123 in PBS (pH 7.4) was added to the *Candida* cell at a final concentration of 2 mg/mL and incubated in the dark for 30 min. Subsequently, the cells were collected by centrifugation at 4,000 rpm for 5 min, thoroughly washed twice with PBS and again resuspended in PBS. As a control, untreated cells were used. A spectrofluorometer (Shimadzu UV-1700, Shimadzu, Kyoto, Japan) was used to evaluate the fluorescence intensity at 480 nm for excitation and 530 nm for emission.

### Potassium leakage

The protocol of Watanabe et al. (2006) was adapted with minor amendments to estimate extracellular K+ leakage. Briefly, *Candida* cells were inoculated in 100 mL SDB medium and incubated in a shaking incubator for 18 h at 30°C at 120 rpm. *Candida* cells were collected after 5 min at 5,000 rpm, washed three times with sterile PBS buffer, and again suspended in 10 mL of PBS buffer. The dry cell weight was calculated using 500 µL of cell suspension. Then 2 mL of *Candida* cell suspension was mixed with 2 mL of PBS buffer containing different concentrations of Fr. 3 (0.5X MIC, MIC and 2X MIC) and incubated for 4 h at various time intervals (30, 60, 120 and 240 min). *Candida* cells treated with clotrimazole were used as a positive control, whereas untreated *Candida* cells served as a normal control. Following treatment, the cells were removed by centrifugation for 10 min at 10,000 rpm, and the supernatant was collected and preserved for the analysis of the extracellular K+ content released into the medium. Using a Flame Photometer, the potassium filter was used to estimate the K+ concentration (Evans Electroselenium LTD., Halsted Essex, England).

### Leakage of DNA and RNA through the *Candida* membrane

The leakage of cellular contents was assessed based on the method described by Tao et al. (2014), with slight modifications. Briefly, *C. albicans* was suspended in YPD to achieve a final concentration of 1 × 106 cells/mL. To determine the concentration of the released constituents after 0, 30, 60, and 120 min of treatment with various concentrations of Fr. 3, 50 µL of supernatant was collected by centrifugation. Then the supernatant was diluted 1:10 with PBS and filtered through a syringe filter (0.22 mm), and the leakage of cellular materials was assessed by measuring the absorbance at 260 nm with the help of a BioPhotometer (Eppendorf D30, Eppendorf, Germany). *Candida* cells treated with clotrimazole were used as a positive control, whereas untreated *Candida* cells served as a normal control. The experiment was conducted in triplicate.

### Analysis of extracellular pH

To determine the extracellular pH of *C. albicans* after Fr. 3 treatment, 100 µL of *Candida* suspension (105 CFU/mL) was added to 20 mL of SDB and incubated for 48 h at 37°C. After centrifuging the samples at 3,000 rpm for 15 min, the *Candida* cell pellet was collected and washed three times with double distilled water. After that, the *Candida* cells were again resuspended in 20 mL of sterile double distilled water and treated with various concentrations of Fr. 3. The level of extracellular pH in the samples was determined at 0, 30, 60 and 120 min, using a Systronics µpH system 362 pH meter. Samples without Fr. 3/clotrimazole were used as controls, whereas samples treated with clotrimazole alone were used as positive controls.

### Cytotoxicity study

The cytotoxicity of the Fr-3 was determined on horse red blood cells by testing the hemolytic activity using the method described previously, with some modifications (Ahmad et al., 2010). Briefly, 5 mL of horse blood was centrifuged in a sterile 15 mL falcon tube at 2000 rpm for 10 min. The supernatant was discarded, and the cell pellet was washed three times with chilled PBS solution. The final cell pellet was used to yield a 10% RBC suspension with chilled PBS. The 10% RBC suspension were further diluted to 1:10 in PBS, then 100 μL of this suspension was added to different concentration of Fr-3 (0.5× MIC, MIC and 2× MIC) in 1.5 mL tubes. The tubes were then incubated for 60 min at 37°C followed by centrifugation at 2000 rpm for 10 min. From each tube, 200 μL of the supernatant was transferred to a flat-bottomed 96-well microtiter plate and absorbance was monitored at 450 nm spectrophotometrically using a microplate reader (Bio-Rad laboratories, CA, United States). Triton X-100 (1%) and PBS were used as positive and negative controls respectively. The percentage of hemolysis was calculated by the following equation:

$$\%\;hemolysis=\left[\frac{A\;450\;of\;test\;compound\;treated\;sample-A\;450\;of\;test\;buffer\;treated\;sample}{A\;450\;of\;1\%\;Triton\;X\;100\;treated\;sample-A450\;of\;buffer\;treated\;samp}\right]\times 100$$

### Statistical analysis

All experiments were carried out in triplicate, and the values provided were the mean with standard deviation from three different observations for each experiment. The change of data between the treated groups and controls was carried out by applying the Simple Analysis of Variance (ANOVA) followed by Tukey test (95% confidence interval), using SPSS andp < 0.05 was considered significant.

## Results

### Fractionation of the extract

The column chromatography of hydroalcoholic extract of *C. bonduc* seeds yielded several fractions. Column fractions with identical TLC profiles were combined to yield five sub-fractions (Fr 1-Fr 5) as follows: Fr 1 (1–9, 50 mg); Fr 2 (10–15); Fr 3 (16–22, 250 mg); Fr 4 (23–30, 417 mg); Fr 4 (31–37, 156 mg); Fr 5 (38–42, 81 mg).

### Phytochemical screening

Phytochemical screening of Fr. 3 purified from the hydroalcoholic extracts of *C. bonduc* seeds collected from Ernakulam region was carried out using various chemical assays in order to identify either the presence or absence of secondary metabolites such as alkaloids, flavonoids, steroids and triterpenoids. Triterpenes and steroids were relatively frequent in the Fr. 3 of hydroalcoholic extracts.

### Anticandidal activity of the fractions obtained from hydroalcoholic extract of *C. bonduc* seeds

The collected five fractions were separately tested for anticandidal activity against the three test *Candida* species. Here, Fr. 3 recorded significant activity against the tested *Candida* species. *Candida albicans* recorded the best activity against Fr. 3 (8 μg/mL), followed by *C. glabrata* (16 μg/mL) (Table 2). Fr. 5 also recorded activity against the test fungi. As C. albicans recorded the best activity in the anticandidal assay, it was selected for further detailed experiments to determine the mechanism of action.

**TABLE 2:** MIC/MFC of fractions against the three *Candida* species.

Samples	MIC/MFC (µg/mL)		
	*C. albicans*	*C. tropicalis*	*C. glabrata*
Fr. 1	2000/4,000	4,000/8,000	4,000/4,000
Fr. 2	^a^	-	-
Fr. 3	8/16	32/64	16/32
Fr. 4	1,000/2000	-	4,000/8,000
Fr. 5	64/125	250/500	125/250
Clotrimazole	0.5/1	0.25/0.5	0.25/0.5

^a^ Recorded no activity up to 8,000 μg/mL.

### Identification of bioactive compounds in the Fr. 3 by LC-Q-TOF-MS

**FIGURE 1:**
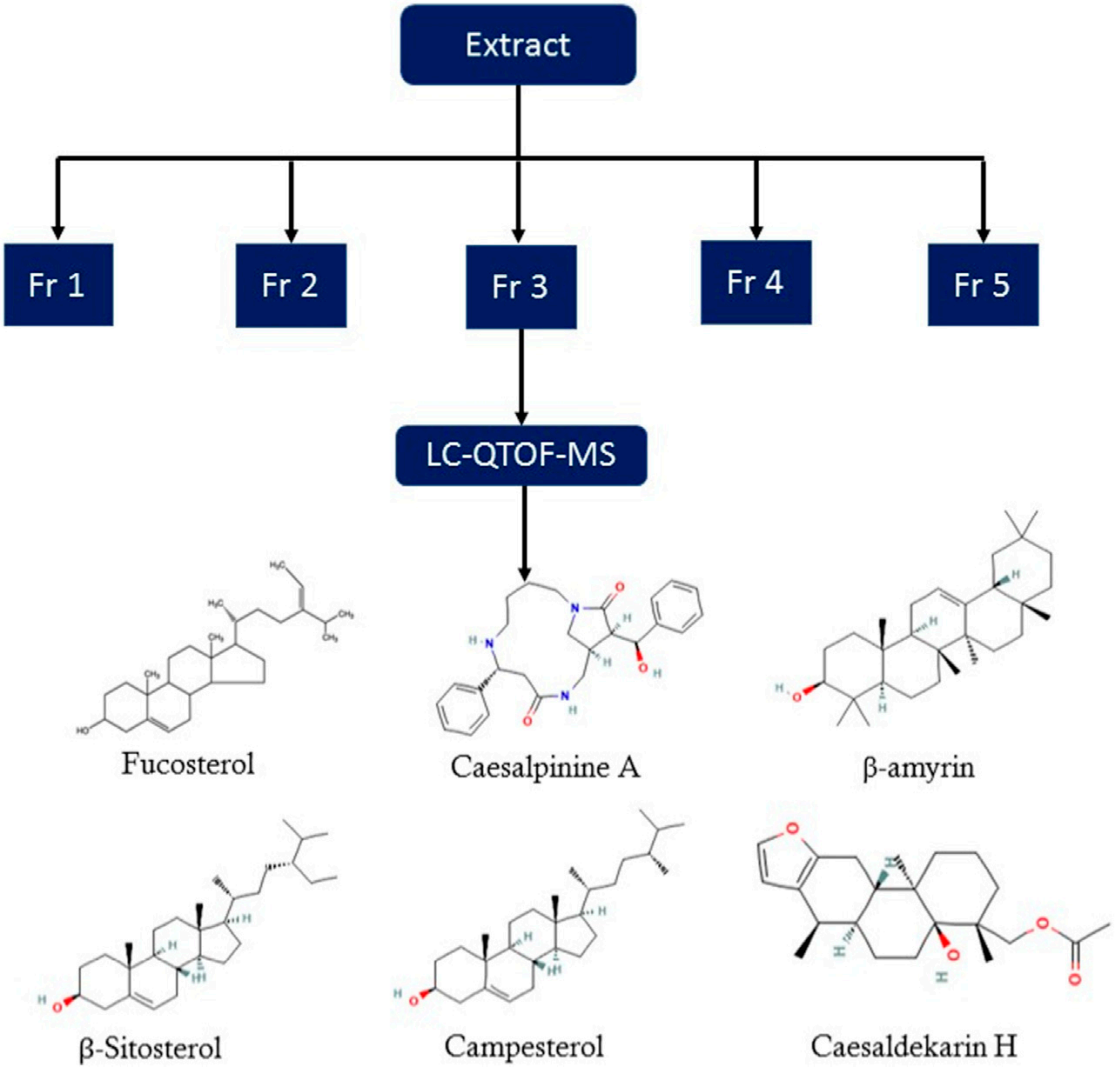
LC/MS-QTOF investigation revealed the presence of various compounds in the Fr. 3.

In the anticandidal assay, Fr. 3 recorded significant activity. For the detection of phytoconstituents in the Fraction, Fr-3 was subjected to LC-Q-TOF-MS analysis which resulted in the identification of 6 phytocompounds, namely, Fucosterol, Caesalpinine A, β-amyrin, β-Sitosterol, Campesterol, and Caesaldekarin H (Figure 1; Table 3). The molecular formula, molecular weight, chemical structure and retention time is given in Table 3.

**TABLE 3:** LC-Q-TOF-MS data of Fr.3

Cpd	Name	Formula	Structure	RT	Molecular weight	Score
1	Fucosterol	C29H48O	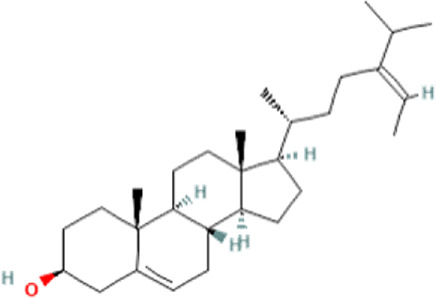	19.721	412.3682	97.64
2	Caesalpinine A	C25H31N3O3	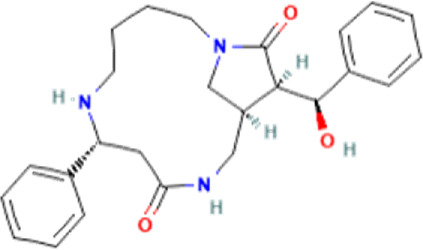	14.934	421.2367	69.19
3	β-amyrin	C30H50O	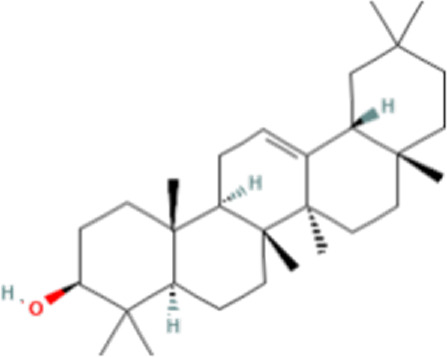	17.429	426.3819	67.34
4	β-Sitosterol	C29H50O	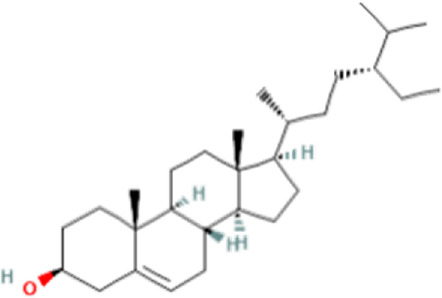	18.973	414.3927	55.91
5	Campesterol	C28H48O	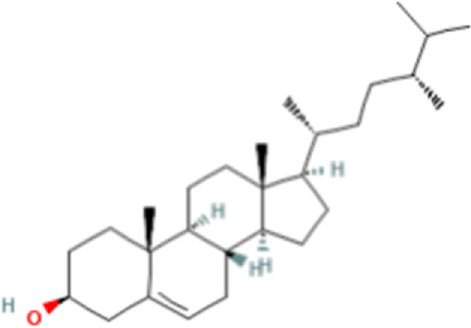	18.658	400.3709	72.72
6	Caesaldekarin H	C22H32O4	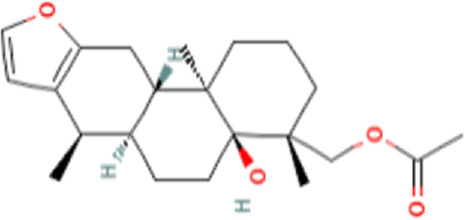	15.402	360.2273	55.26

### Identification of bioactive compounds in the Fr. 3 by GCMS

The GC-MS analysis data of the Fr. 3 is shown in Table 4 which clearly exhibited multiple phytochemical components. The phytochemical composition, retention time, base peak (m/z), chemical class, molecular mass, and formula are also described in Table 4. A comparison of the MS constituents with the NIST library confirmed the identity of five phytochemicals.

**TABLE 4:** Phytochemical detected in Fr. 3 extract identified by GCMS.

No	RT	Name	Formula	MW	Molecular structure	Area %
1	18.975	4H-pyran-4-one,2,3-	C6H8O4	144	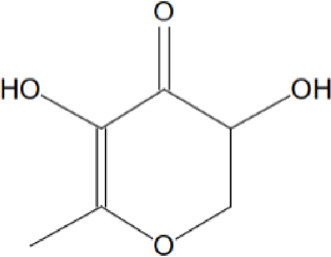	6.729
		dihydro-3,5, dihydroxy- 6-methy-				
2	58.077	n-Hexanoic acid	C16H32O2	256	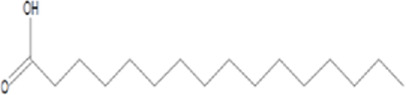	11.062
3	58.332	6-octadecenoic acid	C18H34	282	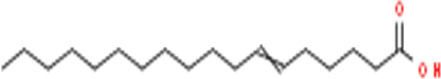	4.331
4	64.772	1,2 benzene dicarboxylic acid mono (2-ethyl hexyl ester)	C16H22O4	278	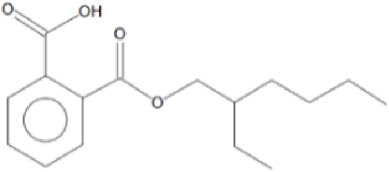	10.826
5	73.242	2′-cyanospiro(1,2,3,4,6,7,12,12b-octa hydroindolo [2,3-day]quinolizine-1,1′ cycloprppane	C25H34O5	414	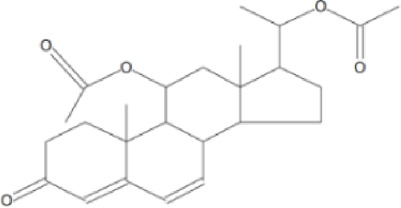	23.226

### Fr. 3 significantly reduced preformed *C. albicans* biofilm

**FIGURE 2:**
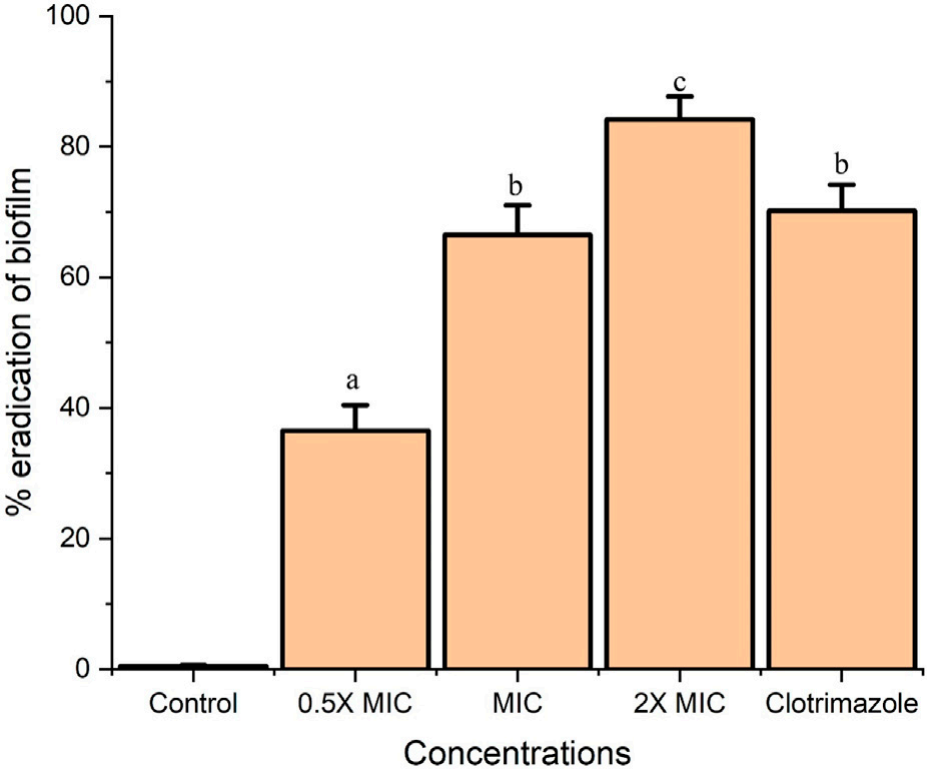
Fr. 3 exhibited dose-dependent inhibition of biofilm in *C. albicans*. The data presented here represent the mean ± SD of triplicate set of experiments. The average followed by different letters are statistically different (*p* < 0.05).

In our experiment, Fr. 3 significantly reduced the biofilms formed by *C. albicans*. From the outcomes, it was recorded that the inhibition of biofilm increases with the concentration of Fr. 3. This clearly demonstrated that the activity of Fr. 3 was dose-dependent. The maximum reduction in biofilm was recorded at 2X MIC concentration (Figure 2). Similarly, standard antifungal agents also reduced biofilm production significantly.

Inhibition of germ tube production.

**FIGURE 3:**
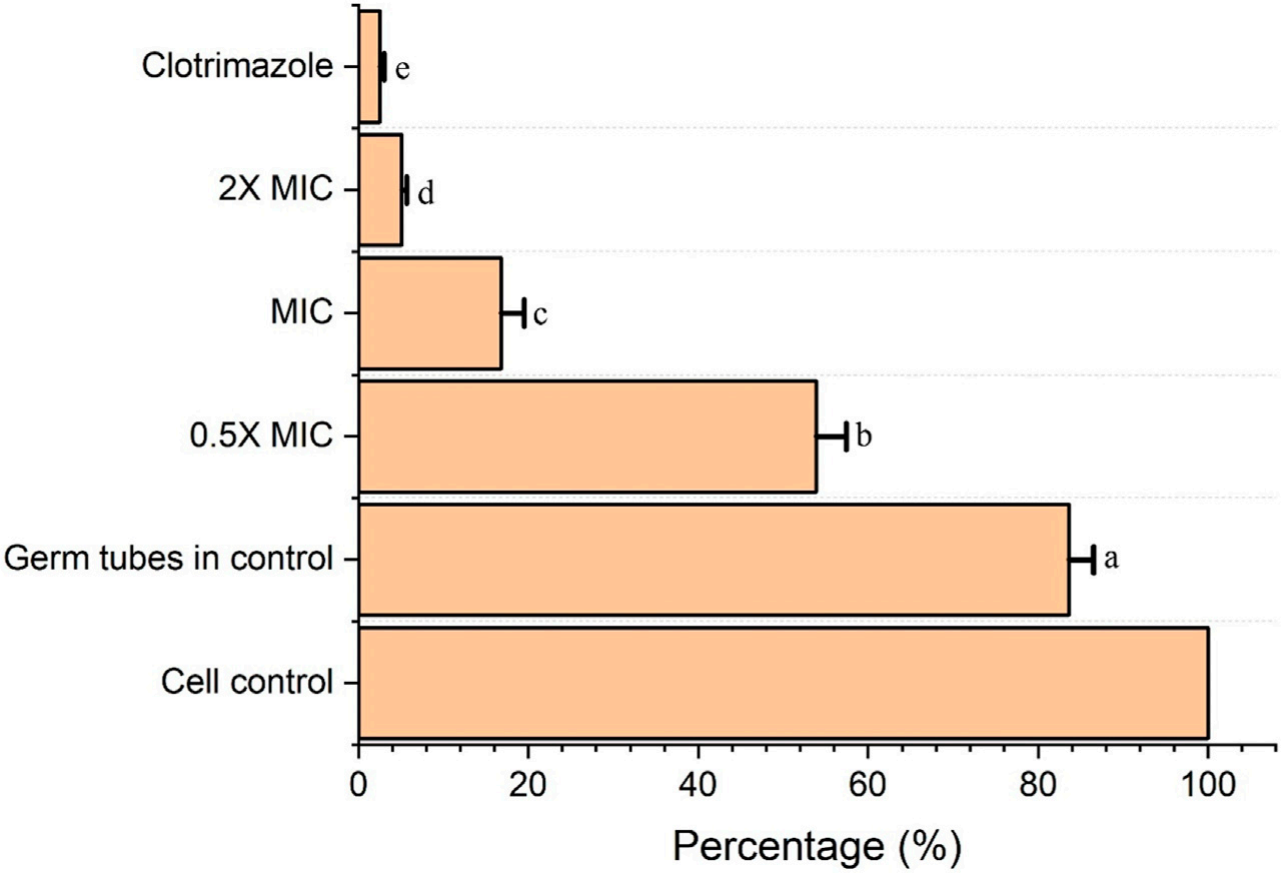
Fr. 3 significantly enhanced the germ-tube inhibition in *C. albicans*. The data presented here represent the mean ± SD of triplicate set of experiments. The average followed by different letters are statistically different (*p* < 0.05).

From the result obtained from the inhibition of germ tube production, it was recorded that the Fr. 3 significantly reduced the germ tube production by *C. albicans* cells. In addition to this inhibition, it increases with an increase in the concentration of Fr. 3. Fr. 3 at MIC and 2X MIC concentrations significantly inhibited germ-tube production (Figure 3).

### Fr. 3 significantly inhibited ergosterol biosynthesis

**FIGURE 4:**
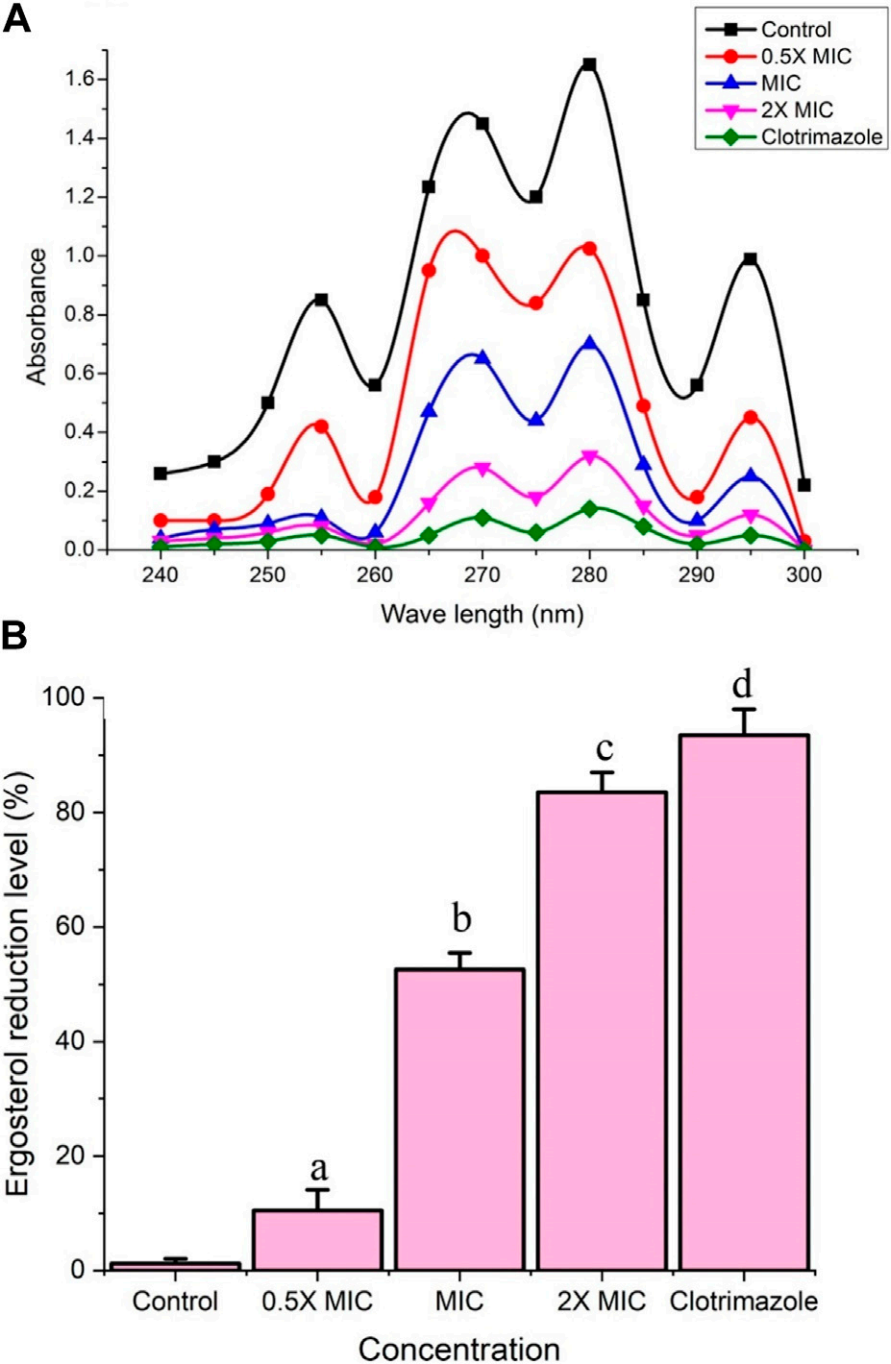
**(A)** The spectrophotometric ergosterol profiles of *C. albicans* after treatment with Fr. 3. Negative control represents the *C. albicans* cells without treatment, whereas the positive control represents *C. albicans* treated with MIC concentration of clotrimazole. **(B)** The reduction percentage of ergosterol levels induced by various concentration of Fr. 3. The data were expressed as mean ± SD. The average followed by different letters are statistically different (*p* < 0.05).

The experiment clearly demonstrated that Fr. 3 significantly affects ergosterol biosynthesis in *C. albicans*. From the results, it was clear that the control sample exhibited the corresponding absorption spectra of the four characteristic peaks of sterols (Figure 4A). However, treatment with the Fr. 3 leads to a substantial reduction in the sterol levels in *C. albicans*. Moreover, when the concentration of Fr. 3 increases, the reduction level of sterols also increases significantly (Figure 4A).

Furthermore, Fr. 3 recorded a significant reduction percentage in the biosynthesis of ergosterol (Figure 4B). Here clotrimazole inhibited the ergosterol biosynthesis almost completely. MIC of Fr.3 resulted in 52.6% inhibition, whereas 2X MIC exhibited 83.5%, (Figure 4B). The results demonstrated that Fr.3 could significantly decrease the intracellular sterols of *C. albicans*, which facilitated the major antifungal action.

### Compounds present in the Fr. 3 recorded excellent binding to lanosterol 14-α-demethylase in molecular docking studies

**FIGURE 5:**
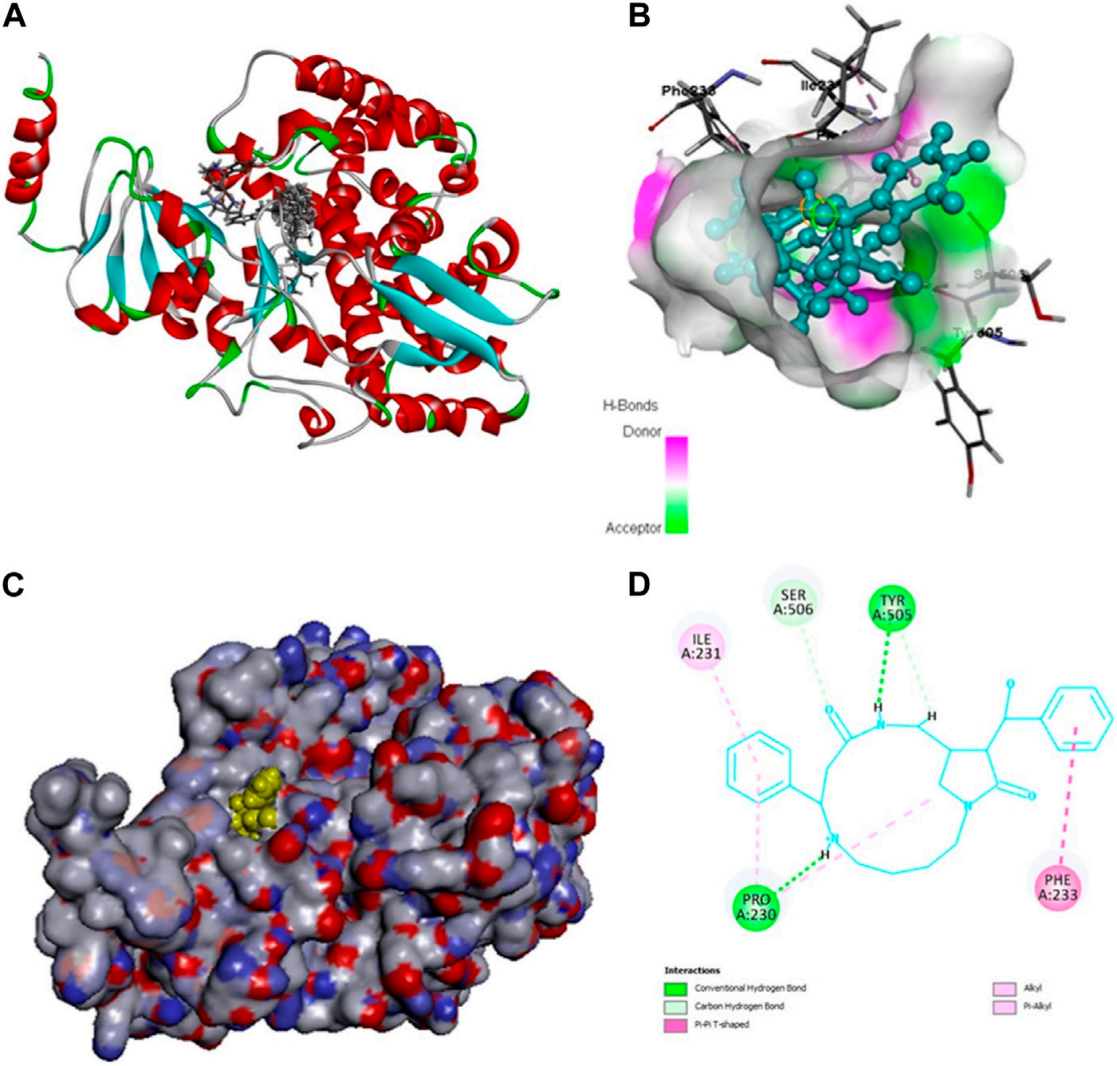
**(A, B)** Observed interactions between Caesalpinine A, Fucosterol, β-sitosterol, Campesterol, Caesaldekarin H and Lanosterol 14-α-demethylase. 5FSA Docked with Caesalpinine A, Fucosterol, β-sitosterol, Campesterol, Caesaldekarin H. **(C, D)** 5FSA Docked with top scoring ligand Caesalpinine A.

The bioactive compounds found in Fr.3 fit into the active site of the Lanosterol 14- α -demethylase enzyme [Protein Data Bank (PDB) ID: 5FSA], according to molecular docking analysis. This enzyme is absolutely required for fungal plasma membrane biosynthesis and maintenance (Figure 5). The binding affinity between the various phytochemicals present in Fr. 3 and the target protein (LibDock score) and the associated binding energies are shown in Table 5.

**TABLE 5:** Docking Results of various chemicals present in Fr. 3 against Lanosterol 14-α-demethylase by LibDock.

Index	Name	LibDock score	Binding energy	Ligand energy	Protein energy	Complex energy
1	Caesalpinine A	121.28	3491.07	3820.32	−32140.1	−24828.7
2	Fucosterol	100.99	35637.1	1067.16	−32140.1	4564.17
3	β-sitosterol	100.449	2316.17	192.812	−32140.1	−29631.1
4	Campesterol	94.464	302.362	160.573	−32140.1	−31677.1
5	Caesaldekarin H	79.2802	2617.37	174.748	−32140.1	−29348

Figure 5 represents the outcome of the various bioactive compounds present in Fr. 3 docked with the Lanosterol 14-α-demethylase enzyme. Here in the docking study, Caesalpinine A recorded best docking against 14-α-demethylase enzyme (LibDock score: 121.28) and the binding energy of the enzyme coupled with Caesalpinine A (3491.07 kcal/mol), showing hydrogen bonds with cyclic N-H group with residues TYR505 and PRO230 while steric interaction was observed with residues PHE233, ILE231 and PRO230 as given in Table 6. The docking study was in correlation with the ergosterol inhibition assay, thereby validating their antifungal activity. Out of six compounds from Fr. 3, β-amyrin failed to dock at the 14-α-demethylase enzyme.

**TABLE 6:** Interaction between lanosterol 14-α-demethylase with Caesalpinine A.

Name	Distance	Category	Types
5458904-Caesalpinine A: H33 - A: TYR505: O	2.16242	Hydrogen Bond	Conventional Hydrogen Bond
5458904-Caesalpinine A: H34 - A: PRO230: O	2.94039	Hydrogen Bond	Conventional Hydrogen Bond
A: SER506: HA - 5458904-Caesalpinine A: O3	2.79983	Hydrogen Bond	Carbon Hydrogen Bond
5458904-Caesalpinine A:H42 - A: TYR505: O	3.0986	Hydrogen Bond	Carbon Hydrogen Bond
A: PHE233–5458904-Caesalpinine A	4.86257	Hydrophobic	Pi-Pi T-shaped
5458904-Caesalpinine A - A: LEU87	4.95334	Hydrophobic	Alkyl
5458904-Caesalpinine A - A: PRO230	4.17609	Hydrophobic	Pi-Alkyl

Observed interactions between 14-alpha demethylase and standard antifungal agents such as itraconazole, ketoconazole, miconazole, and fluconazole were shown in Supplementary Figure S1. Among these, the best binding affinity was shown by itraconazole with a score of−11.6 kcal/mol mainly by hydrogen bonding with TYR132 (Supplementary Figure S2).

### Analysis gene expression revealed that Fr.3 down regulate the ERG11

**FIGURE 6:**
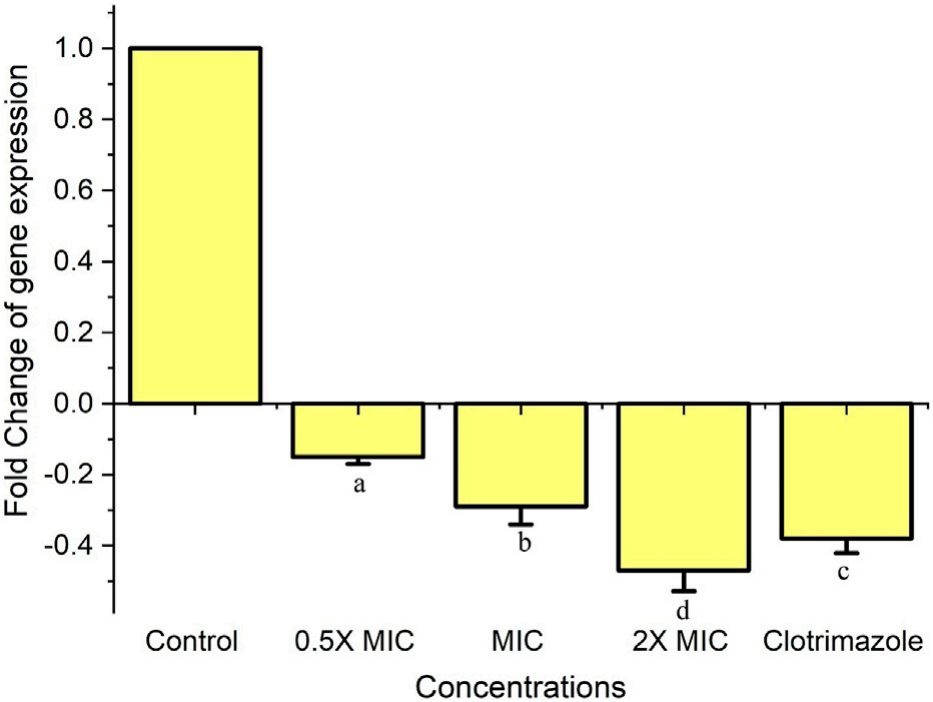
Expression of ERG11 gene in C. albicans treated with Fr. 3. Real-time RT-PCR was used to determine the relative abundance of the ERG11 gene in C. albicans after 24 h of treatment with different concentrations of Fr. 3. The data are means of fold changes with standard error from three separate experiments that were replicated three times. The data represents average ± standard deviation values. The average followed by different letters are statistically different (*p* < 0.05).

In this study, *C. albicans* was treated with Fr. 3 and clotrimazole. The expression levels of ERG11 in *C. albicans* cells were monitored by quantitative real-time RT-PCR. ERG11, the gene responsible for the synthesis of Lanosterol 14-α-demethylase, was found to be significantly downregulated (*p* < 0.05) in *C. albicans* cells exposed to various concentrations of Fr. 3 (Figure 6). The gene expression patterns of *Candida albicans* were similar in response to the antifungal activity of MIC concentrations of Fr. 3 and clotrimazole. ERG11 expression levels showed the lowest fold change in expression in *C. albicans* treated with 2X MIC concentration of Fr. 3. DNA sequencing was used to confirm the authenticity of the PCR products. The sequences revealed a high degree of homology to related genes in the Gene Bank.

### Treatment with Fr. 3 enhances the accumulation of ROS in *C. albicans*

**FIGURE 7:**
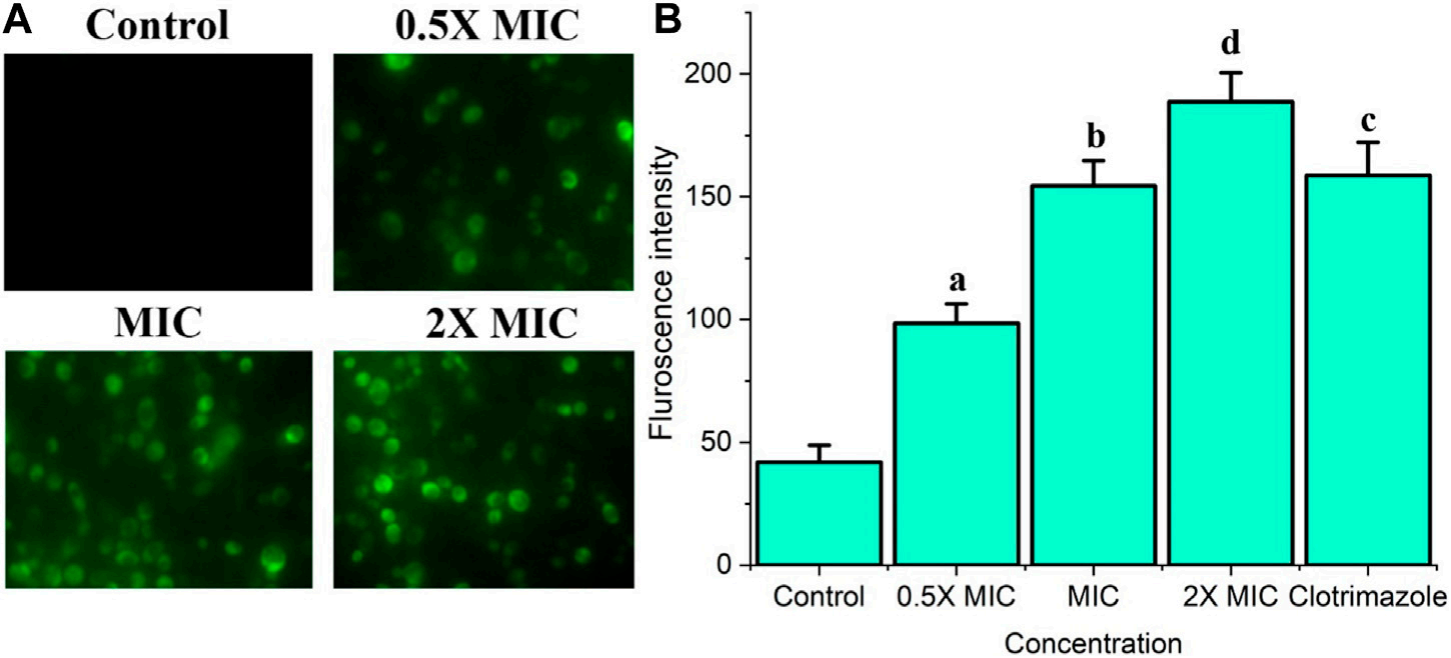
The effect of Fr. 3 on intracellular ROS generation. *Candida* cells were exposed to increasing concentrations of Fr. 3. After staining with DCFHDA, the samples visualized by fluorescent microscope and fluorescent intensity was calculated using a spectrophotometer. **(A)** The product of ROS in the *C albicans* cells treated with Fr. 3 detected by fluorescence microscopy. **(B)** Fluorescent intensity observed in spectrophotometer. The graph represents average ± standard deviation values. The average followed by different letters are statistically different (*p* < 0.05).

The generation of ROS by Fr. 3 was quantified using DCFH-DA staining and indicated by DCF fluorescence due to DCFHDA probe oxidation in *C. albicans*. Fr. 3 revealed a dose-dependent progressive increase in the intracellular ROS levels under the tested conditions (Figure 7). This finding suggests that the Fr. 3-treated cells experienced oxidative stress and ROS accumulation.

### Fr. 3 disturbs *C. albicans* plasma membrane

**FIGURE 8:**
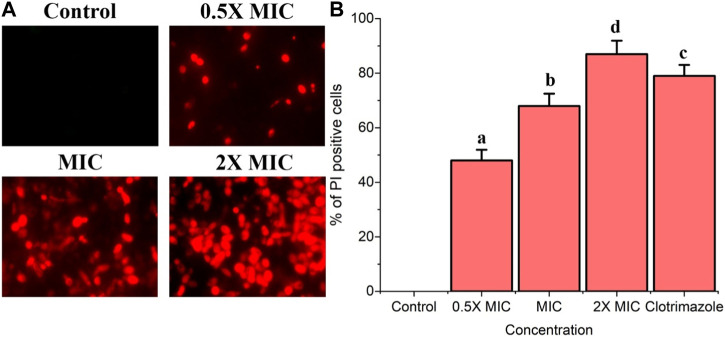
The integrity cell membrane of *C. albicans* was assessed using PI staining. The PI uptake assay revealed that Fr. 3 has a significant impact on the membrane integrity in *C. albicans*. **(A)** Fluorescence microscopy image of membrane damage as evidenced by red *Candida* cells after Fr. 3 treatments. **(B)** The proportion of PI-positive *Candida* cells after treatment with different concentrations of Fr. 3. The percentage of PI-positive *Candida* cells was calculated by counting PI-positive cells against normal cells in 10 different fields. The graph depicts the average ± SD values. The average followed by different letters are statistically different (*p* < 0.05).

In order to confirm the impact of Fr. 3 on *Candida* cell membranes, the integrity of the cell membrane was analyzed using PI staining. In PI staining studies, Fr. 3 resulted in the progressive elevation of the PI stained *Candida* cells in a dose dependent manner (Figure 8A). As shown in Figure 8B, PI staining analysis revealed a significant red fluorescence signal in Fr 3-treated cells compared with control cells, indicating that Fr. 3 disrupted *C. albicans* cell membrane integrity.

### Effect of Fr. 3 on cell membrane potential

**FIGURE 9:**
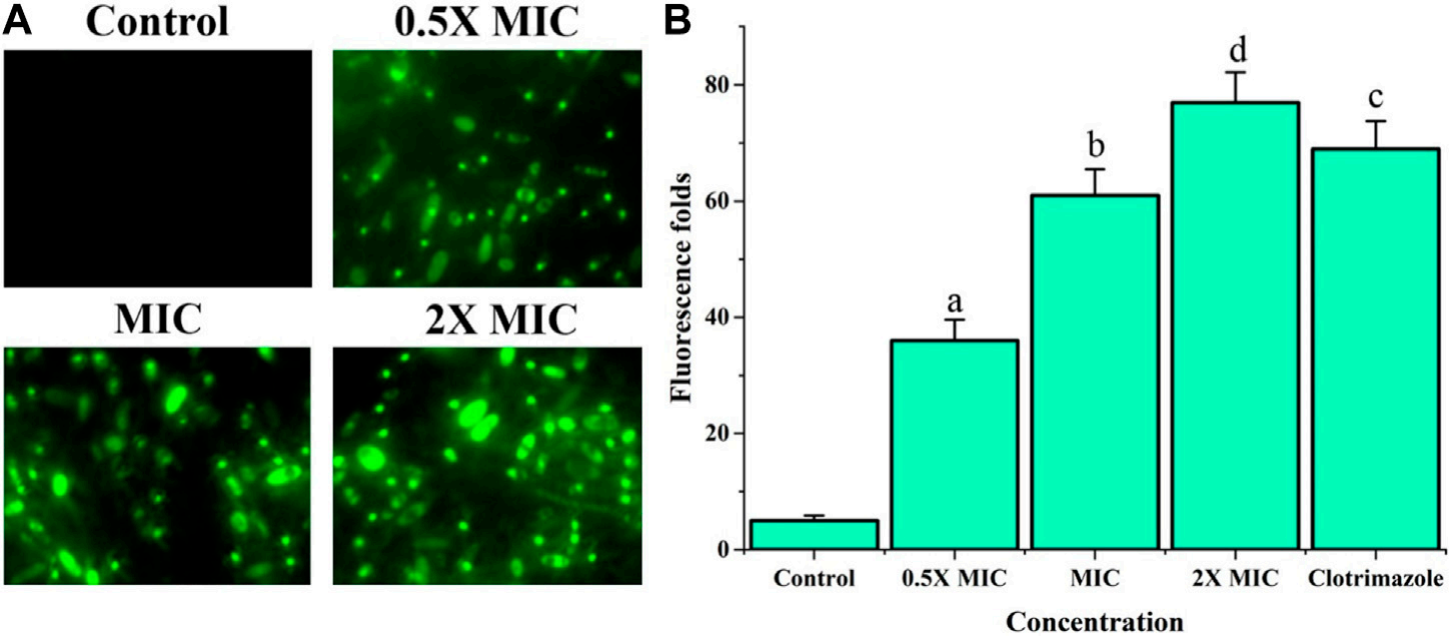
Impact of Fr. 3 on cell membrane potential of *C. albicans*. Rho 123 assay recorded that *C. albicans* treated with Fr. 3 significantly induced the cell membrane potential. **(A)** Fluorescence microscopy image Rho 123 uptake *Candida* cells as evident by the intense bright green colored after Fr. 3 treatment. **(B)** Spectrofluorophotometric investigation of the enhancement in relative fluorescence intensity of *C. albicans* cells after Fr.3 treatment. The graph depicts the average ± SD values. The average followed by different letters are statistically different (*p* < 0.05).

When *C. albicans* was exposed to Fr. 3 at different concentration, the membrane potentials also changed significantly based on enhancement in the mean fluorescence fold of Rho 123, a specific cationic lipophilic fluorescence dye (Figure 9A). Based on the outcome, it was clear that Fr. 3 at 2X MIC concentration significantly induced the membrane potential of C. albicans compared with control (Figure 9B).

### Potassium leakage

**FIGURE 10:**
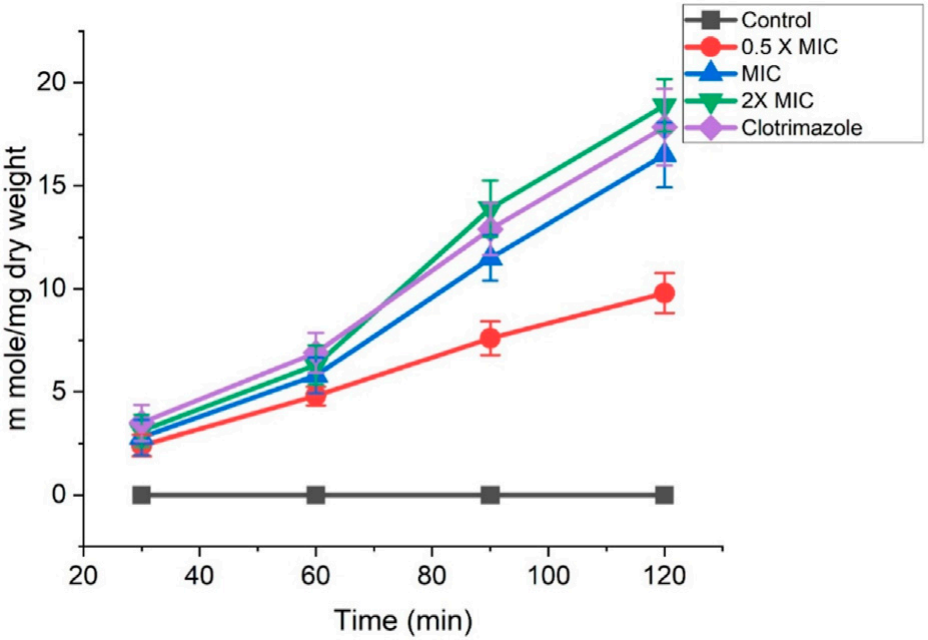
Effects of Fr. 3 on the extracellular K+ leakage in C. albicans. The graph represents average ± standard deviation values.

Fr. 3 recorded significant leakage of potassium ions from the *C. albicans*. At 2X MIC, Fr.3 leads to maximum leakage of K+ from the *C. albicans* (17.85 m mol/mg dry weight of cell). In addition to this, leakage of K+ increases with increase in the concentration of Fr. 3 (Figure 10). The results were statistically significant at *p* ≤ 0.05.

### Fr. 3 induced significant leakage of DNA and RNA through the *C. albicans* cell membrane

**FIGURE 11:**
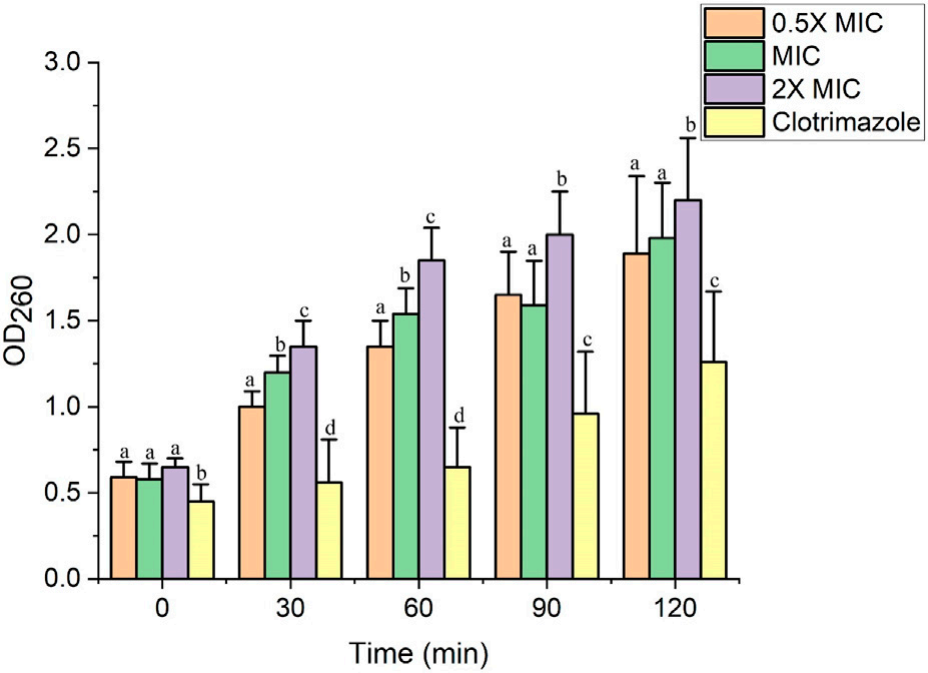
The impact of Fr. 3 the release of DNA/RNA from the C. albicans. The graph represents average ± standard deviation values. The average followed by different letters are statistically different (*p* < 0.05).

The effect of Fr. 3 on *C. albicans* membrane degradation and cellular constituent release was determined at 0, 30, 60, and 120 min after treatment, and the outcomes are depicted in Figure 11. *C. albicans* exposure to Fr. 3 causes a significant (*p* < 0.05) increase in the release of genetic materials, which increased with exposure. In comparison to the untreated control sample, the intracellular compounds were released earlier after 30 min of exposure to Fr. 3. *Candida* cells treated with clotrimazole showed minimal and consistent cellular release.

### Treatment with Fr. 3 causes significant increase in the extracellular pH

**FIGURE 12:**
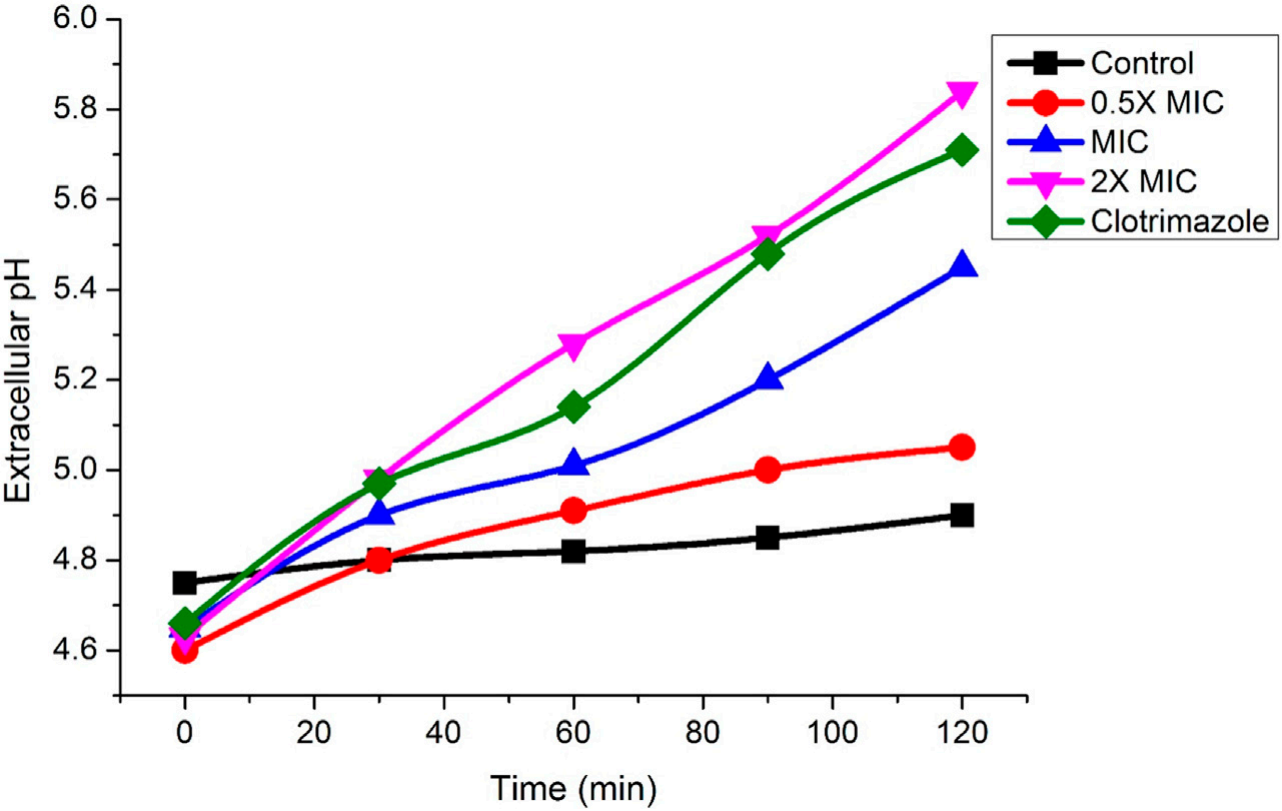
Impact of Fr. 3 on the increase of extracellular pH in *C. albicans* treated. The graph represents average ± standard deviation values.

Figure 12 depicts extracellular pH measurements of *C. albicans* treated with Fr. 3 and untreated cells. As demonstrated in the figure, *C. albicans* treated with Fr. 3 showed an early substantial increase in extracellular pH when compared to control cells that were not treated. Clotrimazole-treated *C. albicans* also had higher extracellular pH values than controls. As a result, these findings confirm that Fr. 3 causes fungal cell membrane damage.

### Cytotoxicity studies by hemolytic activity

**FIGURE 13:**
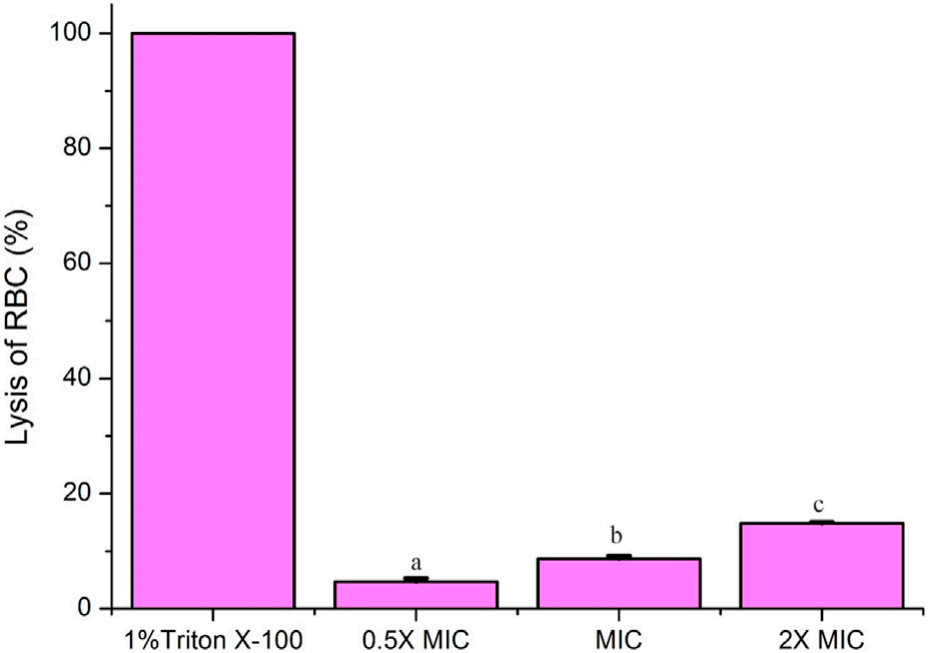
Cytotoxicity assay by hemolytic activity. Fr-3 at varying concentrations showed significant difference in hemolysis of RBCs compared to positive control 1% Triton X-100 (reference for 100% hemolysis). PBS was used as negative control, which showed complete absence of hemolysis. Data are presented from three independent experiments using means ± S.D. The average followed by different letters are statistically different (*p* < 0.05).

Cytotoxicity of Fr. 3 at varying concentrations was checked by hemolytic assay using horse red blood cells (RBCs). When compared to positive control (Triton X-100), which causes 100% lysis, while Fr. 3 recorded a mild hemolysis at 2X MIC (Figure 13). PBS was used as a negative control, which showed no lysis of RBCs. These results confirmed significantly low cytotoxicity effect of Fr.3, suggesting that they are potentially safe to use.

## Discussion

The prevalence and mortality rates of various fungal infections have elevated significantly in the last few years, especially candidiasis caused by *Candida* spp. (Guevara-Lora et al., 2023). Although *C. albicans* is the most common pathogen responsible for candidiasis, nonalbicans *Candida* spp. such as *C. glabrata*, *C. krusei, C. dubliniensis, C. parapsilosis*, and *C. tropicalis* are also major opportunistic fungal pathogens responsible for causing various hospital-associated infections. These pathogens are also a high risk for immunocompromised patients undergoing various surgeries or chemotherapy (Guevara-Lora et al., 2023). C. albicans is a major opportunistic human pathogen that can reside in the oral cavity, intestinal tract, and vaginal area as a commensal microbiota. When patients are subjected to long-term broad-spectrum antibiotics, chemotherapies, or have weakened immune systems, they develop both superficial maladies and potentially fatal disseminated infections (Yue et al., 2022). Furthermore, C. albicans can easily form biofilm, making these organisms difficult to treat. Azole drugs were widely used to treat *Candida* infections, but they are now completely ineffective in treating *Candida* biofilms, necessitating the development of new drugs to manage the infection effectively. On the other hand, unlike antibiotics, developing antifungal drugs is extremely difficult due to the fact that yeasts are eukaryotic organisms with structural similarities to human cells. As a result, new antifungal drugs are more likely to cause unwanted side effects. As a result, there is a pressing urgency to investigate new antifungals that are extremely beneficial, safe, and bearable. Natural products, particularly extracts and phytochemicals from medicinal plants, are regarded as a treasure trove for drug discovery. In the present study, we investigated the anticandidal activity of various fractions purified from the hydroalcoholic extract of *C. bonduc* seeds.

In this study, first we fractionated the hydroalcoholic extract of *C. bonduc* seeds. After fractionation, similar fractions were pooled on the basis of TLC. The pooled fractions were then subjected to a preliminary anticandidal assay through MIC studies. In the MIC assay, Fr. 3 recorded the best activity and was thus selected for detailed investigation to elucidate the mechanism of action. Subsequently, Fr. 3 was subjected to two different fingerprinting techniques, i.e., LCMS and GCMS analysis for detecting the presence of various phytochemicals in the fraction. LCMS and GCMS analysis revealed the presence of terpiniods types of compounds (Heinrich et al., 2022).

Subsequently, the Fr. 3 was subjected to the *C. albicans* biofilm inhibition assay. *Candida* biofilm is a systematic structure composed of planktonic and mycelial yeast forms that is surrounded by extracellular polymeric materials. Furthermore, biofilms are a morphological form of several microbial strains that are highly resistant to antimicrobial substances when compared to their planktonic equivalents (Bassyouni et al., 2019). As a result, antimicrobials capable of inhibiting the planktonic form of microorganisms should be tested for their outcome on biofilms of the same organism. Plants are abundant in bioactive molecules with diverse biological and pharmaceutical properties. As a result, in recent years, numerous types of investigations have focused on new clinical approaches with the support of natural phytocompounds with antibiofilm potential from medicinal plants to manage the consequences associated with biofilm forming pathogens. The extracts of several medicinal plant species have antifungal/antibiofilm activity against pathogenic *Candida* spp. (Shao et al., 2016). Furthermore, extracts from powerful medicinal plants can inhibit or eliminate biofilm formation at concentrations greater than 99% at 1 mg/mL (Shao et al., 2016). In the present study, the inhibition of biofilm-producing ability of *C. albicans* was investigated with various concentrations of Fr. 3 and the result was clearly evident that Fr. 3 destroyed the pre-formed biofilms significantly. In this study, a 2X MIC concentration of Fr. 3 was able to inhibit 84.2% of biofilm eradication. The biofilm eradication (%) obtained here was comparable to that obtained with clotrimazole, a drug known to be effective against *Candida* biofilm. Given the above findings, additional research will be conducted in the future to elucidate the mechanism underlying C. albicans biofilm inhibition.

*C. albicans* has a variety of morphological forms, including yeast cells, germ tubes, true hyphae, and pseudohyphae. The first stage in the formation of true hyphae by *Candida* spp. is the formation of germ tubes from blastoconidia. This characteristic is very vital for the pathogenesis *Candida* because it is responsible for the penetration and invasion of epithelial cells, which cannot occur during the blastoconidia stage. Intracellular germ tube formation allows the *Candida* to escape from the immune cells of phygocyted pathogen. Hence, this dimorphic transition is one of the most vital virulence factors associated with *Candida* spp. (Stojanović et al., 2016). As a result, reducing or blocking germ tube production in order to control candidal infections would be a significant accomplishment. In this study, the germ-tube-producing ability of C. albicans under the treatment of various concentrations of Fr. 3 was investigated. Here, Fr. 3 significantly inhibited the germ tube production of C. albicans. Numerous investigations are carried out worldwide to inhibit the morphological transitions in *Candida* spp. Unfortunately, little is known about the mechanism of action of natural compounds against the virulence trait exhibited by *C. albicans* (Bravo-Chaucanés et al., 2022). A few reports are available on the effect of natural and synthetic compounds against germ tubes or pseudo-hyphae produced by *Candida* spp. (Bravo-Chaucanés et al., 2022).

Next, we investigated the effect of Fr. 3 on the ergosterol content of *C. albicans*. Changes in ergosterol content would add to our understanding of ergosterol biosynthesis inhibition. Ergosterol is a major component of the fungal plasma membrane and is accountable for cell membrane integrity. In this study, Fr. 3 significantly reduced ergosterol content by interfering with cell membrane integrity. Furthermore, ergosterol inhibition increases as the concentration of Fr. 3 increases. Previously, natural and synthetic compounds were used to reduce ergosterol in *Candida* species in a dose-dependent manner. The outcome of our investigation further supports the dose-dependent inhibition of ergosterol biosynthesis that was previously reported (Ahmad et al., 2011; 2015). Conventionally used antifungals, such as azoles and polyenes, target the ergosterol biosynthesis pathway of fungi. In addition to these two proven antifungal classes of drugs, this pathway and the enzymes involved have been widely investigated as a possible drug target for finding novel antifungal drugs (Suchodolski et al., 2021). Because of the evolutionary connection that exists between human hosts and fungal pathogens, drugs make differentiating between human and fungal cells difficult. Hence, ergosterol has become a major target for antifungal drug development since it is not present in the human host. Besides that, ergosterol is an essential element of the fungal cellular membrane and has received a lot of attention because of its vital contribution to preserving the physiology, integrity, fluidity, and rigidity of *Candida* cells (Abe & Hiraki, 2009). Furthermore, ergosterol has been shown to coordinate membrane heterogeneity and prevent water penetration in fungal cells (Abe and Hiraki, 2009). From this, it was clear that ergosterol played a pivotal role in the survival of *Candida* spp. Hence, the inhibition of ergosterol has been used as a vital target in drug development.

The ergosterol biosynthesis pathway has been recognized as a target of azole drugs via the inhibition of enzymes and its associated genes like lanosterol 14-demethylase (ERG11), squalene epoxidase (ERG1), and sterol C-14 reductase (ERG24)/sterol C-8 isomerase (ERG2) (Rather et al., 2022). As a result, the enzymes and genes involved in the biosynthetic pathway of ergosterol are very important for the drug discovery programme for the pathogenic fungal species. In addition to this mutation of genes associated with the biosynthetic pathway of ergosterol are directly linked to severe drug resistance in *Candida* spp. [8]. Out of several genes, ERG11, associated with the biosynthetic pathway of ergosterol, is very important. Thus, any compounds targeting ERG11 lead to a rise in lanosterol levels, causing further damage to the cell membrane and integrity (Rather et al., 2022). In our investigation, we found Fr. 3 significantly downregulating ERG11. The ergosterol quantification results show the impact of this gene’s downregulation. In addition to this, the downregulation of the ERG11 gene by Fr. 3 is comparable with the result of clotrimazole.

The ergosterol inhibition may result in the damage of the cell membrane, which then leads to the loss of various ions from the *Candida* cells, affecting the osmotic balance. Changes in potassium ions (K+) can easily disrupt a cell’s osmotic balance. Moreover, the ion leakage from a cell can lead to cell lysis. Because ions play critical roles in the activation of essential enzymes, which act as biological catalysts that mediate several vital biochemical reactions (Arya et al., 2010). Thus, we have investigated the effect of Fr. 3 on K+ leakage in C. albicans cells and the efficacy was compared to that of clotrimazole. From the result, it was clear that Fr. 3 recorded significant K+ leakage in C. albicans. The information obtained from the Fr. 3 clearly suggested the alteration of the *C. albicans* membrane, which further increases its permeability to release K+ ions. The phospholipids, sphingolipids, and sterols that make up the fungal plasma membrane serve as a permeability barrier while small molecules are transported. Among other things, these components transmit signals and serve as a matrix for proteins with various functions (Oehlschlager and Czyzewska, 1992; Georgopapadakou and Tkacz, 1999). Due to this, when the membrane is damaged, a tremendous increase of efflux in intracellular K+ happens. Cox et al. (2000) investigated the effect of tea tree oil on *C. albicans* and concluded that leakage of K+ was a clear sign of cell membrane damage. Likewise, Wei et al. (2008) investigated plasma membrane injury in *C. albicans* by analysing K+ leakage and concluded that K+ outflow was associated with membrane permeability. As a result, it can be concluded that Fr.3 increases cell membrane permeability, inhibits ergosterol synthesis, or causes membrane damage in C. albicans cells.

Oxidative stress, particularly ROS-induced oxidative stress, was thought to be a vital disorder that promotes cell death in organisms. Furthermore, excessive ROS damage a variety of biomolecules, including nucleic acids, proteins, and lipids. These damages can eventually lead to cell death in addition to increasing cell membrane permeability (Hata et al., 2010). In this study, we looked into the induction of ROS by Fr. 3 in *C. albicans*. According to the findings, Fr. 3 induced quick and momentous ROS generation in *C. albicans* (Figure 7). Next, we performed a PI uptake assay to see if the fungicidal effect of Fr.3 is related to its ability to disrupt fungal membranes. Based on the outcome of the PI uptake assay, it was clear that Fr. affects membrane integrity and increases membrane permeabilization of *C. albicans*. The fluorescence intensity was measured after being treated with various concentrations of Fr. 3 using PI staining. The increased fluorescence in Fr. 3 treated cells indicated increased membrane permeability, which reveals the cell membrane damage in *C. albicans* (De Castro et al., 2015). Potassium release assays revealed even more damage to the membrane’s permeability.

Damage to the plasma membrane is also reported to cause the downfall of the electrochemical potential because of the formation of pores (Qin et al., 2011). The formation of pores leads to a loss of osmotic balance, which leads to the entry of ions and liquids into the cytoplasm of the cells. In addition to this, pores may lead to leakage of cytoplasmic contents like proteins, carbohydrates, and ribonucleic acids, resulting in cell death (Taveira et al., 2018). Fr. 3-treated *Candida* cells released significant amounts of intracellular material (at 260/280 nm) in our study. The obvious loss of intracellular protons confirmed the compromised permeability of the plasma membrane in Fr. 3-treated *C. albicans*. This also results in an increase in extracellular pH, which was also evident from our investigation.

Cell membrane potential is a vital component that plays an important role in ATP production and metabolic activity in the cell. The alteration in membrane potential is thought to be one of the quickest responses of cells to external stimuli (Curtin et al., 2002). Rho 123 analysis conducted in *C. albicans* treated with Fr. 3 revealed a significant increase in the fluorescence intensity when compared to the control. This clearly indicated that Fr. 3 could hyperpolarize mitochondrial membrane potential. As a result, the change in membrane potential is one of the mechanisms of antifungal activity. ROS are produced in the mitochondria as well. Excess ROS causes lipid peroxidation of the mitochondrial membrane, which alters membrane performance and, ultimately, membrane potential (Shao et al., 2016). As a result, an increase in reactive oxygen species and a change in membrane potential are the causes of mitochondrial damage.

From our investigation, it was clear that Fr. 3 demonstrated two mechanisms of action, i.e., inhibition of ergosterol biosynthesis and production of ROS. But the major mechanism of action of Fr. 3 is inhibition of ergosterol biosynthesis, which was proved by both *in vitro* and *in silico* analysis. Earlier, several compounds were reported to demonstrate multiple mechanisms of action in *Candida*. Silva et al. (2020) reported that Punicalagin, a phenolic compound extracted from Lafoensia pacari, triggers ergosterol biosynthesis disruption and cell cycle arrest in Cryptococcus gattii and *C. albicans*. This clearly pointed out that punicalagin has multiple mechanisms of action in *C. albicans*. Similarly, honokiol, a bioactive polyphenol, a major active ingredient in Magnolia officinalis, also demonstrated multiple modes of action in *C. albicans* (Sun and Liao, 2020). In their study, honokiol decreased the ergosterol content and upregulated the expression of genes related to the ergosterol biosynthesis pathway. Furthermore, honokiol caused abnormalities in vacuole morphology and function. In addition to this, honokiol also disrupted the intracellular calcium homeostasis of *C. albicans* via the calcineurin signaling pathway (Sun and Liao, 2020). Shao et al. (2016) also demonstrated that berberine is a bioactive herbal-originated alkaloid that attenuates *C. tropicalis* via ROS increase, ergosterol decrease and efflux inhibition.

## Conclusion

**FIGURE 14:**
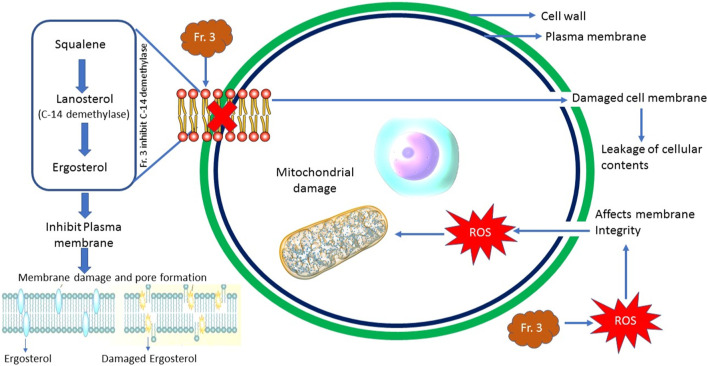
Overview of antifungal modes of action exhibited by Fr. 3 against *C. albicans*.

In sum, we identified that Fr. 3 purified from the hydroalcoholic extract of *C. bonduc* seeds, displayed promising antifungal activity against pathogenic *C. albicans*. The mechanism investigations revealed that Fr. 3 interferes with the ergosterol biosynthesis, which inhibits the cell growth of *C. albicans*. The *in silico* study exposed that phytochemicals identified from Fr. 3 are a promising inhibitor of the enzyme 14-α-demethylase, a major enzyme associated with the biosynthesis of C. albicans cell membrane (Figure 14). Our investigation further recorded the antifungal potential of Fr. 3, which is being linked with ROS production, which leads to the damage of the plasma membrane. In addition, Fr. 3 is very effective in inhibiting fungal biofilms and hyphae production (Figure 14). Thus, the outcome of the study clearly shows that Fr. 3 has more than one cellular target in its antifungal potential. As a result, this investigation clarifies expectations for long-term *in vitro* and *in vivo* pharmacological and toxicological research with Fr. 3, with the goal of developing a therapeutic application against *Candida* spp. infections that could aid in the reduction of drug resistance in fungal pathogens.

## Data Availability

The raw data supporting the conclusion of this article will be made available by the authors, without undue reservation.
