# Supplementary material for: A semi purified hydroalcoholic fraction from Caesalpinia bonduc seeds causes ergosterol biosynthesis inhibition in Candida albicans resulting in cell membrane damage
Source: Front Pharmacol. 2023 Jun 12;14:1189241. doi: 10.3389/fphar.2023.1189241 (PMC10291067; doi:10.3389/fphar.2023.1189241)
Supplement: Supplementary file 1 [file Table1.DOCX]

**Supplementary data**

**A semi purified fraction (Fr.3) from hydroalcoholic extract of Caesalpinia bonduc seeds Target** **14**α**–Demethylase (ERG 11) Enzyme causing ergosterol biosynthesis inhibition leading to cell membrane damage in *Candida albicans***

**Shan Sasidharan^1*^, Kumar S Nishanth^1^, Hareendran Nair J^2^**

^1^Department of R&D, Pankajakasthuri Herbal Research Foundation, Pankajakasthuri Ayurveda Medical College Campus, Killy, Kattakada, Trivandrum, Kerala, India, Pin-695572

^2^Department of R&D, Pankajakasthuri Herbals India Pvt. Ltd., Pankajakasthuri Ayurveda Medical College Campus, Trivandrum, Kerala, India

*Corresponding Author:

Pankajakasthuri Herbal Research Foundation,

Pankajakasthuri Ayurveda Medical College Campus, Trivandrum

Kerala, India

e-mail: drshansasidharan@yahoo.co.in

**Molecular docking of 14-alpha demethylase protein with Azole drugs**

**Material and methods**

Multiple ligand molecular docking of the ligands and target proteins was performed byAutoDock Vina (using the PyRx GUI. The structures of Linosterol 14-alpha demethylase (PDB ID: 5FSA) with a structural resolution of 2.16 Å were downloaded from the RCSB PDB Protein Data Bank (https://www.rcsb.org) in PDB format. The prepared protein was then imported into PyRx (Trott and Olson, 2010). Although the active sites of the protein are known, blind docking was performed in which the entire protein was searched for possible ligand binding sites, since alternative sites are also possible sites for potential inhibitors, changed into pdbqt format and set as the receptor protein. For the structures of each compound, we searched them in the PubChem database (https://pubchem.ncbi.nlm.nih.gov) for their PubChem CID/SID number and 3D structures. We obtained each compound structure in the standard Simplified Molecular-Input Line-Entry System (SMILES) SDF file format. All ligand sdf files were imported and converted into PDBQT format. A grid box was defined with dimensions (x = 98.437, y = 15.038, z = 57.689) and a number of points (x = 72.530, y = 81.105, z = 92.698). The exhaustiveness was set to its default (8). The docked poses with the lowest binding energy and root mean square deviation (RMSD) were selected. All molecules were ranked according to their predicted docking score, which was based on the empirical free energy function. The Discovery Studio visualizer, programmed and developed by Accelrys, provides extensive information about ligand-receptor interactions. The two-dimensional and three-dimensional interaction images of docked proteins were obtained.

**Results**

Observed interactions between 14-alpha demethylase with molecules such as itraconazole, ketoconazole, miconazole, and fluconazole were shown **Table 1**. The binding energy of compounds with 14-alpha demethylase was between-7.8 and−11.4, which is considered good. The results for the protein indicated that these compounds are excellent candidates for further research. Only compounds that scored above−7.5 kcal/mol were considered the best ligands among the 4 docked compounds. Among these, the best binding affinity was shown by itraconazole with a score of−11.6 kcal/mol. majorly by hydrogen bonding with TYR132. It inhibits lanosterol 14 alpha-demethylase. Ketoconazole also showed a good binding affinity of −10.3 kcal/mol with 14-alpha demethylase. It shows excellent pharmacokinetic properties. It is miconazole another compound that showed good binding scores of−8.2 kcal/mol. fluconazole also shows a good binding score of−7.8 kcal/mol. hydrogen bonding with LYS90. It blocks ergosterol synthesis by inhibiting the enzyme 14-alpha demethylase (Yoshida, 1988)

**Supplementary Figure S1: Docking results of azole drugs with binding affinity and ligand protein interaction**

| compound | BS (kcal/mol | Ligand-protein interactions | 2D interactions |
| --- | --- | --- | --- |
| Itraconazole  **H- Bond (**TYR132) | -11.6 | 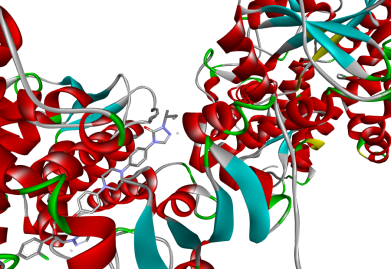 | 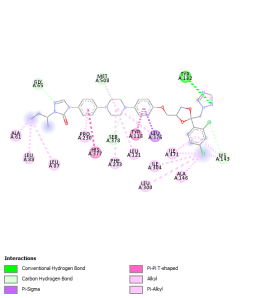 |
| Ketoconazole | -10.3 | 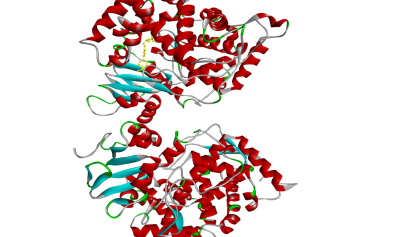 | 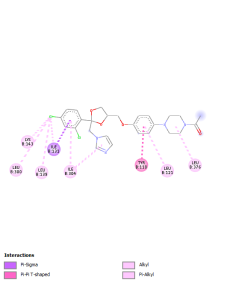 |
| Miconazole | -8.2 | 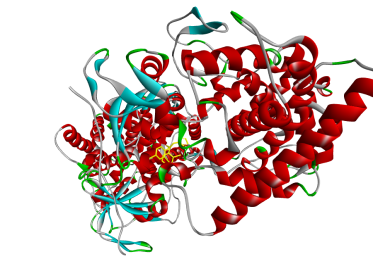 | 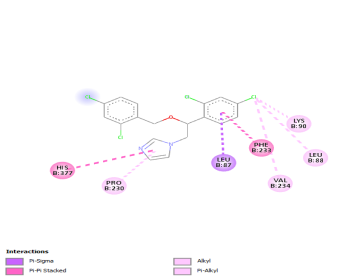 |
| Fluconazole  **H- Bond**  **(**LYS90**)** | -7.8 | 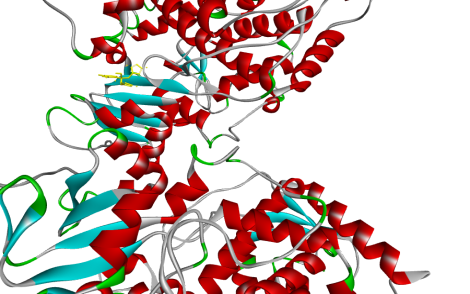 | 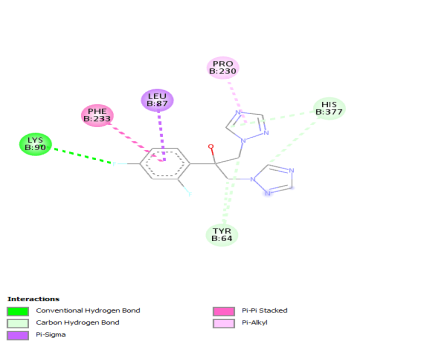 |

**Supplementary Figure S2: Detailed interaction with Itraconazole**

| 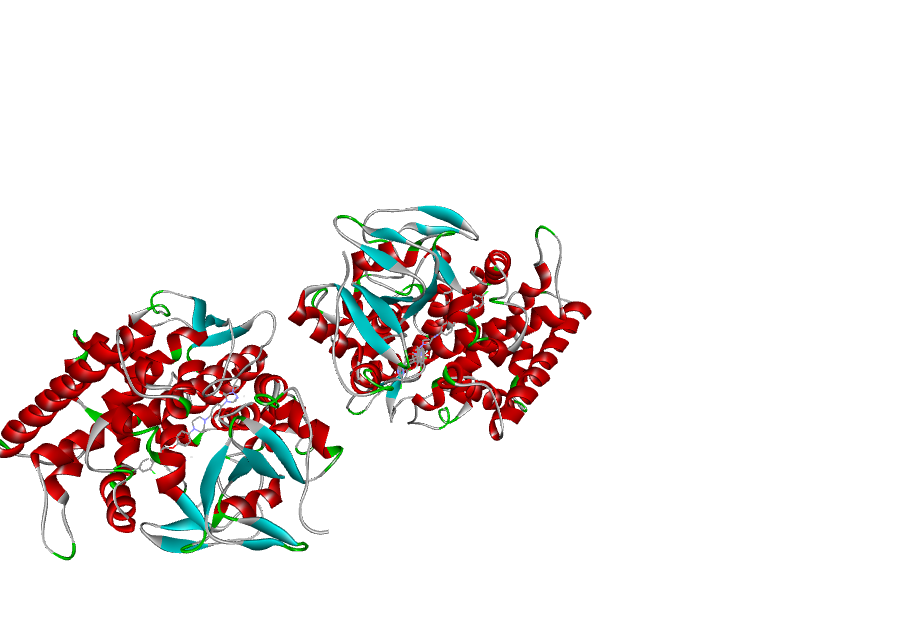 | 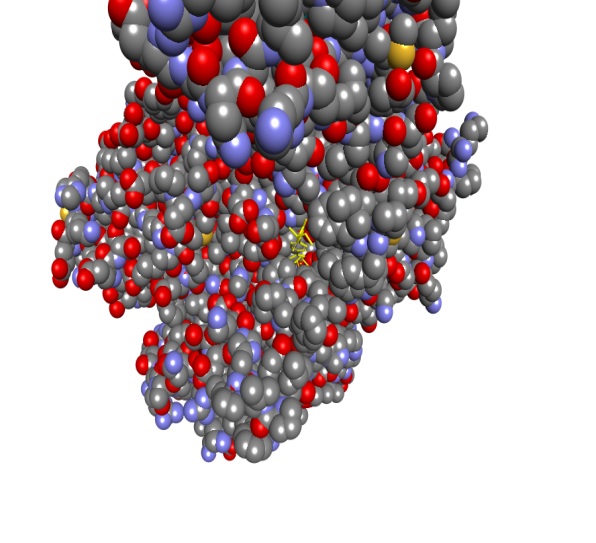 | | 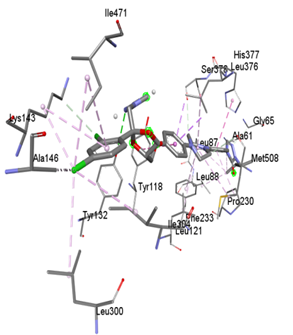 |
| --- | --- | --- | --- |
| 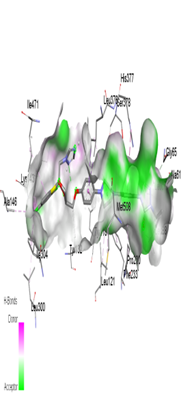 | | 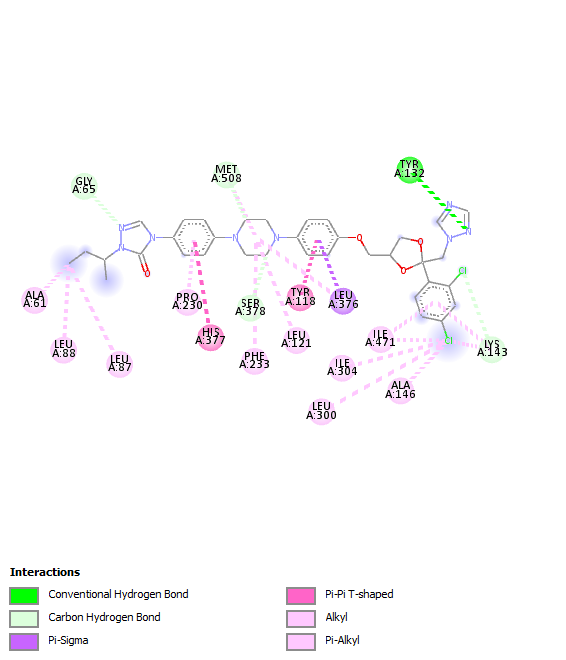 | |
